# A Critical Review of Solid Materials for Low-Temperature Thermochemical Storage of Solar Energy Based on Solid-Vapour Adsorption in View of Space Heating Uses

**DOI:** 10.3390/molecules24050945

**Published:** 2019-03-07

**Authors:** Hao Wu, Fabrice Salles, Jerzy Zajac

**Affiliations:** Institut Charles Gerhardt Montpellier – UMR CNRS 5253, UM, ENSCM, Place E. Bataillon, CEDEX 05, 34095 Montpellier, France; hao.wu@umontpellier.fr (H.W.); fabrice.salles@umontpellier.fr (F.S.)

**Keywords:** energy storage, low-temperature thermochemical storage, space heating uses, solid-vapour adsorption, open sorption systems, adsorption of water vapour, moist-air flow operating mode, porous adsorbents

## Abstract

The present report deals with low-temperature thermochemical storage for space heating, which is based on the principles of vapour adsorption onto solid adsorbents. With the aim of obtaining comprehensive information on the rationalized selection of adsorbents for heat storage in open sorption systems operating in the moist-air flow mode, various materials reported up to now in the literature are reviewed by referring strictly to the possible mechanisms of water vapour adsorption, as well as practical aspects of their preparation or their application under particular operating conditions. It seems reasonable to suggest that, on the basis of the current state-of-the-art, the adsorption phenomenon may be rather exploited in the auxiliary heating systems, which provide additional heat during winter’s coldest days.

## 1. Introduction

The rapidly growing global population together with the general expectation for further improvement of life standards in different parts of the world are generating an unending spiral of increasing consumption of food and use of energy and thus have an enormous impact on the ecosystem. Over the past few decades, the overwhelming conviction has emerged that planning and operation of energy supply and energy usage are essential to keep the balance between the necessity of assuring permanent access to energy for the greatest possible proportion of the world population at affordable costs and the objectives of resource conservation, climate protection, and cost savings. As a notable example, the European Community has launched the “Europe 2020 strategy” including ambitious targets for reductions of greenhouse gas emissions, the use of renewable energy, and energy efficiency [1]. The research and development of a renewable energy plan has become imperative to tackle these environment and development issues, especially in relation with the use of energy in the building sector which is at a critical state in many developed countries [2,3,4,5] (e.g., building applications account for over 40% of total energy usage and 36% of CO_2_ emission in the European Union). Furthermore, the greatest part of this energy usage in buildings is connected with space heating (e.g., over 60% in 2015 according to the French Environment and Energy Management Agency, ADEME [6]), and a long way ahead of electrical devices and domestic hot water supply. Several important initiatives have been taken by regional authorities to promote low-energy buildings using active solar and passive solar systems both to harness the energy and to reduce the overall energy usage. Converting sunlight into heat and electricity remains certainly one of the most efficient solutions to reduce greenhouse gas emissions to safer levels in numerous countries located in regions of high annual solar radiation, e.g., in China, Japan, United States, India, or some parts of the European Union [7,8,9,10]. Nevertheless, in many regions around the world, important fluctuations of energy supply due to the intermittent nature of such a renewable energy source, progressing decentralization of energy conversion and supply, as well as the time and rate mismatch between supply and demand of energy may have serious repercussions on the short- and long-term effectiveness of the implemented solar technologies. In consequence, there is a growing need for adequate energy storage systems so as to solve many of the specific problems related to the energy management.

The existing technologies for short-term thermal energy storage in building applications are mature and robust enough to manage the daily mismatch between the energy supply and demand [11,12,13,14,15,16,17]. For any method of thermal storage, high-temperature solar energy is used in the initial step (i.e., *charging step*) to drive the underlying reactions or phenomena in the endothermic direction. The energy stored in the products is released on demand (i.e., *discharging step*) by lowering the temperature of the storage system and thus driving the reactions or phenomena in the exothermic direction. The selection of appropriate materials is a determinative point, which is done according to some common criteria, the most important being: (i) high energy density (i.e., the energy released in one charging-discharging cycle by a unit volume or mass of the working material), (ii) minimum deterioration in performance or degradation of the working material with repeated cycling, mostly related with the thermodynamic reversibility of the underlying reactions or phenomena, as well as the thermal stability of the material. Depending on the particularity of the storage method and construction of the storage unit, the choice is usually made for low-cost and non-toxic materials with low volume and weight. The sensible heat storage based on the heat capacity of the working material and the latent heat storage making use of a material which stores heat while changing phase or dissolving in an aqueous solution have been implemented industrially on large scale [18]. Nevertheless, they may be accompanied by a great loss of energy and costly investment in thermal insulation (c.f., Table 1).

Seasonal solar energy storage for space heating is an important challenge because the storage unit should store energy for many months (e.g., summer-winter cycle changeover) and release it without much loss. Since the investment costs of high-performance thermal insulation are prohibitive, thermochemical storage using chemical potential changes may be considered as the best compromise solution [14]. Theoretically, energy stored as chemical potential is not subject to thermal loss through the insulation and unlimited charging-discharging cycles can be envisaged. Moreover, most suitable reversible chemical reactions or sorption phenomena produce heat effects which are often one order of magnitude higher than latent heats of fusion and condensation. This category of storage systems is very heterogeneous and includes, among others, sorption phenomena, e.g., adsorption at the solid-gas interface, absorption of vapours in liquids [14], as well as reversible thermochemical reactions, e.g., carbonation or hydration of calcium, strontium, and barium oxides, sulphur-based redox cycles, metal oxide reduction–oxidation or perovskite-type hydrogen production [19,20,21]. The thermochemical reactions where the energy storage principle involves the breaking and forming of chemical bonds usually have high energy densities ranging between 1440 and 3960 MJ m^−3^ (i.e., between 400 and 1100 kWh m^−3^). However, high temperatures (over 773 K) are generally required during the charging step, which makes such storage systems more appropriate for application in solar power plants where the solar heat is used directly to drive the reaction. Furthermore, the process flow diagrams for the energy storage cycle may be quite complex, thus contributing to higher cost and complexity of design for the material containers and auxiliary systems required for storage and delivery of energy.

This review presents the state-of-the-art of research, which associates proposals for the use of solid materials as adsorbents in *low-temperature thermochemical energy storage by adsorption at the solid-gas interface* together with collection of fundamental data on which to base the rationalized approach to materials selection. Although the subject has been already treated in numerous review articles [14,15,22,25,26,27,28], the presentation of potential adsorbents is usually placed in a broader context of thermal and thermochemical storage of energy and, consequently, the particularity of the present case escapes readers’ attention. Other and rarer articles on sorption heat storage focus on more or less advanced system prototypes making use of specific materials in a restricted number [28]. As a result, there is still a need to critically assess the potential candidates by referring strictly to the possible adsorption mechanisms, as well as practical aspects of their preparation or their application under particular operating conditions. The aim is to consider the impact the materials selection would have on the performance and durability of the storage cycle and thus on a successful technology implementation. The necessity of filling this gap has been the main motivation behind the present review. Finally, it has been also decided to put the emphasis on the adsorption of water vapour onto appropriate porous adsorbents, given the fact that this system was by far the most frequently reported case in the scientific literature on the subject, apparently posing less serious security and environmental threats or technical challenges.

From the fundamental point of view, the effectiveness of any heat storage unit mainly relies on reversible thermal phenomena or reactions which support the storage principles. In the context of heat storage by adsorption at the solid-gas interface, it should never be forgotten that adsorption arises due to presence of unbalanced forces at the surface of a solid phase and it can follow various reaction pathways controlled not only by the composition of an adsorption system but also by the experimental conditions applied. Since the phenomenon may switch from one pathway to another under different temperature and pressure regimes, the variability of operating conditions, unavoidable in industrial practice, will affect the adsorption reversibility and thus the quantity of energy exchanged with the surroundings during each discharging step.

Reversible adsorption of vapours onto high surface area solids actually appears to be the most promising alternative for thermochemical energy storage, especially in view of space heating uses. It is certainly worth noting here the first practical use of 7000 kg of zeolite 13X beads in energy storage on district heating net in Munich to store about 4680 MJ (i.e., 1300 kWh) during off-peak hours for the subsequent heating of a school building [13,14]. Two examples of solid materials (i.e., silica gel and zeolite) considered for the purpose of thermochemical heat storage by adsorption are given in Table 1; their physical properties and storage performances are compared with those of representative materials used in thermal energy storage. 

## 2. Adequacy of Adsorption Phenomena in Heat Storage

Depending on the chemical nature of oncoming gaseous molecules (i.e., *adsorbate*) and the surface chemical state of the solid substrate (i.e., *adsorbent*), relatively weak intermolecular forces (mostly of the Van der Waals type) or stronger chemical bonds (e.g., hydrogen bonding, charge-transfer interactions, covalent bonds) operating between the adsorbate molecules and atoms, molecules or ions located at the adsorbent surface are responsible for physical or chemical adsorption at the Solid-Gas interface [29,30,31,32]. Some fundamental differences between chemisorption and physisorption fall within the context of the present energy storage applications.

Physical adsorption is an essentially exothermic phenomenon but it produces lower heat effects (25 kJ released at most per mole of gas adsorbed on the surface) and becomes significant when the gas adsorbs near or below the temperature of gas-liquid phase transition. One notable exception is the case of physical adsorption within micropores (i.e., *micropore filling* mechanism): when such pores are sufficiently narrow (in comparison with the size of adsorbing molecules), the enhancement of adsorption energy may result in higher heat values [33,34]. Physisorption does not require any activation energy and equilibrium is rapidly established provided that the adsorption kinetics is not delayed by the mass transfer in the gaseous phase or diffusion inside the pores of a porous adsorbent. Generally, physisorption is easy to reverse simply by application of heat and/or vacuum (i.e., *desorption*). It should, however, be borne in mind that the phenomenon may become irreversible when the pore space in some mesoporous solids is filled with condensed liquid from the gas phase at higher relative pressures (i.e., *capillary condensation* mechanism).

In contrast to physical adsorption, chemisorption does not diminish rapidly with temperature elevation and it is accompanied by much higher heat effects, often exceeding 100 kJ mol^−1^ due to chemical bonds formed between the adsorbing molecules and the adsorbent surface. In the present context, it should be realised that, in very rare cases, chemisorption may have an endothermic character if it is accompanied by the dissociation of the adsorbed molecules on the solid surface (i.e., dissociative chemisorption) [35,36,37]. Given the nature and strength of adsorbate-adsorbent interactions involved, chemical adsorption occurs only if the adsorbate enters into direct interaction contact with the active surface (i.e., *localized monolayer adsorption on active surface sites*). It usually requires activation energy and is very difficult to reverse by vacuum treatment. In this case, desorption can be induced by a special treatment under severe conditions, e.g., high-temperature heating, ionic bombardment, and reductive or oxidative processes. In consequence, both the chemical structure of the adsorbing gaseous molecules and the adsorption properties of the adsorbent greatly change during adsorption.

The above short discussion on the differences between physical and chemical adsorption highlights a number of important issues that deserve special attention of researchers, engineers and technicians when selecting adsorbate-adsorbent couples appropriate for thermochemical energy storage based on solid-vapour adsorption. Within the same category of adsorption phenomena occurring for given adsorbate and adsorbent, the detailed mechanism and thus the amount of heat released during the discharging step may strongly depend on the experimental conditions applied, mostly the temperature and composition of the gas phase. Furthermore, physical and chemical adsorption phenomena are not exclusive and can occur on the same adsorbent surface in various experimental regimes (e.g., chemical adsorption of basic molecules on surface acid sites of mineral oxides followed by the formation of physically adsorbed multilayer [38,39,40,41]). In addition, when the gas phase is not pure but represents a gas mixture (e.g., water vapour, carbon dioxide and other gases in ambient air), adsorption often becomes a selective process as a result of the competition amongst the gas components for being retained on the adsorbent surface [42,43].

Finally, solid-gas adsorption may be used to underpin the working principle of a heat storage unit provided that the charging step in the storage process will be based on endothermic desorption and the discharging one on exothermic adsorption, according to the following reaction scheme, at fixed temperature, T, and equilibrium gas pressure, p:

(1) $$n_{a}\cdot G-S+Q_{a}\;⇄\;n_{a}\cdot G+S$$

where G and S denote, respectively, the gas and solid components of the working couple, $n_{a}$ is the amount of gas adsorbed on the adsorbent surface, $n_{a}\cdot G-S$ stands for the working couple in a given adsorption state, $Q_{a}$ is the *integral heat of adsorption* measuring the total energy change in both directions of the reversible process; the symbol S on the right hand side of Equation (1) refers to the adsorbent possessing a fully clean surface after a complete desorption of the adsorbate. The integral heat released upon exothermic adsorption is related to the *differential heat of adsorption*, $q_{a}$, defined as a partial derivative of the energy change of the system with respect to the quantity of adsorption (it can be thus regarded as a consequence of adsorbing infinitesimal amounts of gas); hence [44,45]:

(2) $$Q_{a}=\int_{0}^{n_{a}}q_{a}dn_{a}$$

For a selected adsorbate-adsorbent couple performing at a given temperature, the amount of gas adsorbed and the differential heat of adsorption may change when the composition of the gas phase varies during the storage process. An *adsorption isotherm* representing the quantity of gas adsorption at a constant temperature as a non-decreasing function of the equilibrium gas pressure, p, i.e., $n_{a}=n_{a}\left(p\right)$, is the most common representation of adsorption. The differential heat of adsorption is usually plotted against the amount adsorbed, i.e., $q_{a}=q_{a}\left(n_{a}\right)$, and is determined either experimentally by direct calorimetry measurements or numerically from the temperature dependence of the adsorption isotherms (i.e., the so-called van’t Hoff procedure) [31,46,47]. The latter procedure is usually applied to compute the so-called *isosteric heat of gas-phase adsorption* from experimental measurements, on the basis of the following expression [48,49]:

(3) $$q_{a}^{st}=RT^{2}{\left(\frac{\partial lnp}{\partial T}\right)}_{n_{a}}$$

Since the pressure p and temperature T are independent variables in such systems, the pressure may be tuned to hold the amount adsorbed $n_{a}$ constant. It is worth noting here that, under equilibrium conditions, physisorption is sensitive to the energetic heterogeneity of solid surface which manifests itself as a monotonous decrease in the differential heat released when the amount of gas adsorbed increases [50]. On the contrary, chemisorption on the same surface sites is usually characterized by a constant differential heat, and hence by an integral heat increasing linearly with increasing $n_{a}$ value.

In conclusion, the integral heat of adsorption and thus the energy storage density will be controlled by various physical factors which affect the amount adsorbed and differential heat of adsorption in strict relationship with the operative adsorption mechanism: chiefly, temperature, composition of the gas phase, specific surface area and mass of the adsorbent or accessibility of the porous structure of the adsorbent as a function of the adsorbate size and nature. Therefore, *to justify the selection of a new working couple*, adsorption isotherms and the related heat curves should be measured as systematically as possible under real conditions of use.

## 3. Practical Aspects of Heat Storage by Adsorption in Reference to Adsorption Mechanisms

The plant operating conditions under which the heat storage process is implemented may differ, sometimes significantly, from those used when the theoretical storage performance of a given working couple (i.e., adsorbate and adsorbent) has been tested in laboratories. Given the sensitivity of certain adsorption mechanisms to experimental conditions, this difference will inevitably result in altered performance of the real storage system and, therefore, should be taken into account at each stage of the design process.

The main point to be stressed here relates to the way in which adsorption and desorption procedures are carried out. In research laboratories, gas adsorption onto solids is routinely measured under conditions necessary to achieve adsorption equilibrium for each point of the adsorption isotherm in a static volumetric apparatus [32,51,52]. In such measurements, calibrated doses of a single reactive gas are brought into contact with the surface of the previously weighed adsorbent by means of an injection system equipped with a valve, the operating principle of which is based on pressure differences. Prior to adsorption experiment, the process of desorption is accomplished by degassing with the use of a high-performance vacuum pump. Economically speaking, *vacuum degassing is an energy-consuming process and, as such, it is not recommended for the charging step in heat storage units*. Perhaps the most convincing argument against this solution is that, once the degassing operation is completed, the adsorbent itself cannot be efficiently transported to a vacuum chamber or stay in a perfect vacuum inside the reactor during several hours or several months up to the next discharging step; the construction of gas-tight containers would be cost-prohibitive.

Adsorption and desorption procedures under dynamic gas flow conditions in which a non-adsorbing gas is constantly flowing through the adsorbent bed at a given flow rate do not require the use of vacuum and are thus more feasible to implement [32]. In adsorption, the flow of the neutral gas plays the role of a carrier medium for the reactive gas and the adsorption isotherms agree quite well with those obtained by vacuum volumetric measurements on a large variety of samples. In desorption, the desorbed molecules are swept from the adsorption chamber via the continuous flow of inert gas. The partial pressure of the adsorbate released from the adsorbent surface to an inert stream is claimed to decrease in a manner similar to that monitored when applying the vacuum technique [53]. Moreover, a faster degassing is expected since the adsorbate species gain additional energy during collisions with the inert gas molecules striking the solid surface.

Whatever the technique used, simultaneous heating of the solid sample is a very efficient way to increase the degassing rate. Nevertheless, the quantity of exothermic adsorption will decrease at higher temperatures. Therefore, there is no point in applying the same temperature during the charging and discharging steps in order to comply with the reaction scheme given by Equation (1). The thermal energy collected from the sun is exploited during the charging step to purify completely the adsorbent surface. Basically, the temperature of desorption should generally be a compromise between the long-term thermal stability of the adsorbent and the efficiency in adsorbent regeneration. Then, the stability of this temperature will depend on the type of solar thermal collectors, heat exchangers, and heat transfer fluids employed in practice [54,55]. Contrary to the solution applied in solar power plants, mild charging conditions will be searched in heat storage by adsorption because even crystalline inorganic porous solids may undergo different structural and textural changes upon heating, especially when subject to a great number of repeated charge-discharge cycles [56,57,58].

In light of the above considerations, the energy storage density will be thoroughly controlled by the heat of adsorption released during the discharging step under the real experimental conditions applied. In order to avoid heat losses and investment costs in thermal insulation, the discharging step should be carried out at temperatures close to ambient [59,60]. This operating temperature limits the number of gases to be used as adsorbates. For illustrative purposes, physical and chemical properties of several potential candidates have been collected in Table 2. Helium and air, as well as the two major components of air, i.e., nitrogen and oxygen, cannot be physically adsorbed under ambient conditions because of their low critical temperatures. On the contrary, they may be used as neutral carrier gases or even heat transfer media, especially helium which is characterized by a higher thermal conductivity (*conductive heat transfer*) and a comparable heat capacity (*convective heat transfer*). Helium molecules have a small kinetic diameter which enables them to penetrate into smaller pores of porous adsorbents. Nevertheless, the possibility of taking air from the atmosphere for free or at negligible cost is certainly the main advantage of using it as the heat transfer fluid; any leakage from the storage unit will not cause environmental problems. Consequently, the endothermic desorption in the charging step may be performed by making use of ambient air with low levels of critical impurities (e.g., water vapour and carbon dioxide) pre-heated to a given temperature by simple solar air collectors [61,62,63].

From a thermodynamic viewpoint, other gases included in Table 2 are considered as potential candidates for adsorbates in the heat storage units. Polar water, ammonia, and alcohol molecules can be physisorbed on any polar or polarizable surface sites, whereas non-polar CO_2_ with a total static polarizability of 2.86 × 10^−30^ m^3^ in the gas phase [64] can interact via Debye induction or London dispersion forces. In comparison with ammonia and carbon dioxide, higher enthalpies of vaporization for water, methanol, and ethanol suggest a potential for higher energy densities in a storage system based on physical adsorption. On the other hand, ammonia and carbon dioxide are characterized by much higher vapour pressures [65], thereby providing an advantage in terms of increased quantities of adsorption and reduced mass transport limitations. 

According to other data reported in Table 2, some of these gases can be chemisorbed onto solids that possess adequate active sites on the surface. More or less strong chemisorption from the gas phase will occur chiefly through acid-base interactions leading to the formation of surface donor-acceptor adducts, which include hydrogen bonds accompanied or not by a proton transfer (the stronger the H-bond the easier is the proton transfer) or covalent bonds for a typical Lewis acid-base pair. In line with higher proton affinities, molecules of water, ammonia and ethanol represent strong Brønsted bases in the gas phase, capable of being chemisorbed on a solid surface having Brønsted acid sites (e.g., hydroxyl groups on mineral oxides). Simultaneously, they may act as hard Lewis bases, thereby bonding to such hard Lewis acid sites as extra-framework cations Al^3+^, Li^+^, Mg^2+^, Na^+^, or Ca^2+^ (their acid hardness parameters are 45.8, 35.1, 32.5, 21.1, 19.7 eV, respectively [66]), which occur in solids containing compensating cations (e.g., zeolites or clays). As a hard Lewis acid, carbon dioxide will form donor-acceptor adducts with hard Lewis base sites (e.g., extra-framework F^−^, OH^−^, Cl^−^ anions with base hardness parameters equal to 7.0, 5.6, 4.7 eV, respectively [66]). On the other hand, water and alcohols may also exhibit acidic character; the order of decreasing gas-phase acidity is as follows: C_2_H_5_OH > CH_3_OH > H_2_O [67].

**Figure 1:**
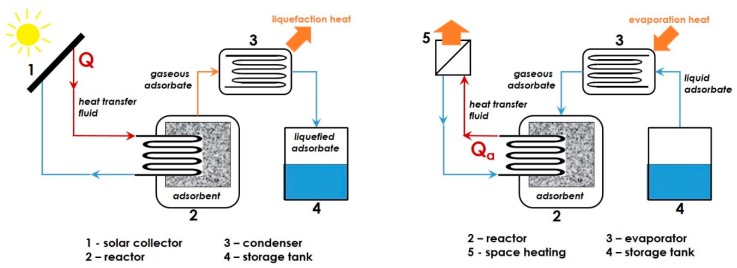
Simplified flow-sheets of the charging (left panel) and discharching (right panel) stages in the low-temperature thermochemical storage of solar energy based on Solid-Vapour adsorption in a closed sorption system.

It should be also mentioned here that the flammability of adsorbate vapours creates potential safety issues and may restrict their use in heat storage. If a given adsorbate has negative health and environmental impacts, or when its contact with air is to be avoided for safety reasons, the heat storage unit should operate in a *closed sorption system* [68]. This technical solution precludes the release of the working gas to the environment and the exchange of thermal energy between the system and its surroundings is realized through appropriate heat exchangers. The gas desorbed from the adsorbent under the action of heat (e.g., via the internal heat exchanger) during the charging step flows into a liquefier, releases energy to the heating system, condenses and is stored on site in liquefied form, as depicted schematically in Figure 1. When the exothermic adsorption is to be carried out in the discharge mode (e.g., during winter), an external power source is necessary to transform the liquefied adsorbate back into vapour. Therefore, the closed storage systems require the presence of two storage tanks, one for the adsorbent and the other for the liquefied adsorbate. Such systems are suitable rather for small-scale applications [14]. 

**Figure 2:**
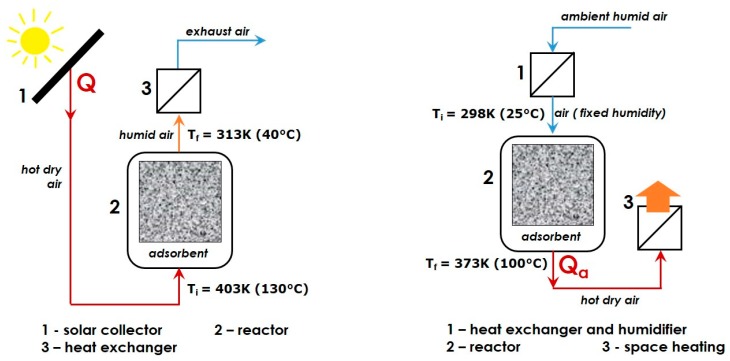
Simplified flow-sheets of the charging (left panel) and discharching (right panel) stages in the low-temperature thermochemical storage of solar energy based on adsorption of water vapour onto solid adsorbents in an open sorption system. Just to give the Reader an indication of the inlet, T_i_, and outlet, T_f_, temperatures at the reactor level, the experimental values have been taken from Ref [75].

Its sheer availability, low cost, non-flammability, and non-toxicity have made water vapour by far the most frequently studied adsorbate for the thermochemical energy storage by Solid-Gas adsorption [27,69,70,71]. In particular, this is the best candidate to be used in *open sorption systems*, together with porous adsorbents possessing a hydrophilic surface [15,63,72]. The temperature of the discharging step should range between 273 and 373 K (i.e., between 0 and 100 °C), unless antifreeze additives are added to or the adsorbate vapour is pressurized. Simplified flow-sheets of the charging and discharging stages are given in Figure 2 for illustrative purposes. During the charging step, the adsorbent is chiefly dried (i.e., endothermic desorption of water vapour) and the heat required for drying is provided to the reactor by an air flow of low relative humidity pre-heated due to harnessing solar energy or another heat source. Then, the dried adsorbent remains in the reactor filled with dry air at ambient temperature for a desired period of time until the next discharge phase. Classically, the adsorption onto previously dried adsorbent is carried out in the “*moist-air flow*” mode by using outdoor air brought inside and additionally saturated with water in order to produce a gaseous flow with a stable humidity rate at a given temperature. The released heat of adsorption renders the air flowing out of the reactor warmer, thereby allowing its direct use for heating. The necessity of pre-heating intake air to the temperature of adsorption certainly reduces the overall efficiency of the storage process. However, this problem may be avoided quite easily by employing an additional heat exchanger where the heat from exhaust gas is transferred to the inlet air, as has been proposed within the framework of the German MONOSORP project [68]. In the particular case of space heating, the adsorbate can be removed directly from the inside of the heated building. The investment costs being lower than those associated with the closed systems, coupled with better heat and mass transfer conditions, as well as lower temperatures required for adsorbent regeneration, are sufficiently compelling arguments in favour of the open adsorption units [73,74]. Nevertheless, working with ambient air may present increased risk of competitive adsorption of water vapour and such major air impurities as CO_2_, likely resulting in the disturbance of the thermal balance underlying the heat storage process.

Finally, the kinetics of adsorption may also affect the efficiency of the storage process. Indeed the rate at which the adsorbate is retained on the adsorbent surface will impact the heat and mass transport during adsorption, in relation with the thermal conductivity and specific heat capacity of the adsorbent used, composition and temperature of the inlet gas, type of the adsorbent bed, as well as the reactor dimensions [13,57,76,77]. With a fixed bed adsorption process in an open sorption system using air as the carrier gas, the temperature distribution at different positions within the reactor and, what is most important in view of space heating applications, the rate of adsorbate depletion in the outflowing air and the temperature variations of the latter are kinetics-sensitive technical characteristics. Appropriate adsorption kinetics resulting in a high thermal power output is considered as having a beneficial effect on the performance of the storage system [77]. On the other hand, such parameters as the volumetric feed-gas flow rate and feed composition, or mass of the adsorbent should be carefully optimised so as to control the length of time during which the total saturation of the adsorbent with the adsorbate is to be achieved, the stability in time of the output temperature, and thus the duration of the heating cycle.

## 4. Comments on the Criteria for Selection of Working Materials as Adsorbents

Given the variety of technical problems associated with the available storage technologies, it is quite difficult to propose clear criteria for the selection of best adsorbents to be used in thermal energy storage by solid-gas adsorption. This difficulty lies in the fact that many of the criteria reported in the literature are contradictory or inconsistent [4,14,21,22]. Rather than wishing to find the perfect adsorbent, it should be admitted that suitable materials and systems depend on the task they have to fulfil under specific conditions.

In a more general sense, the working adsorbent-adsorbate couple has to insure high energy density and high energy efficiency while reducing investment and operating costs and minimizing the environmental issues and risks involved. Based on the considerations made in the two previous sections, good candidates for adsorbents may be evaluated with regard to:

(1) Specific surface area and pore volume for high adsorption capacity towards the selected adsorbate,
(2) Affinity between the active surface of the adsorbent and the adsorbate for high heat of adsorption,
(3) Selectivity towards a given adsorbate when adsorbed from a gas mixture,
(4) Ability to provide appropriate mass transport and kinetics of adsorption,
(5) Thermal conductivity and specific heat capacity for good heat transfer from/to the adsorbent bed,
(6) Ease of regeneration, thermal and chemical stability, and usable lifetime under operating conditions for long-term resistance to repeated cycles of charging and discharging,
(7) Toxicity, environmental impact, corrosiveness, flammability, and compatibility with materials of construction,
(8) Cost, availability, ease of handling, ease of shaping and up-scaling.

The above list is certainly not exhaustive, but it gives an idea of the needs and possibilities for optimising materials design. Some critical remarks about the general strategy for materials preparation and optimisation are given below.

The intra-particle porosity is one of the principal characteristics of the adsorbents that are pertinent to their use in thermochemical energy storage. The adsorption phenomena should be carried out making use of mesoporous (pore size in the range of 2–50 nm) or/and microporous (pore size less than 2 nm) solids in order to enhance the adsorbate uptake and intensify the heat exchange during each charge-discharge cycle [16,57,69,70,77,85,86,87,88,89,90,91,92,93,94]. This is the best way to increase the specific surface area, in line with the first criterion. Indeed, the specific surface areas of meso- and microporous adsorbents are in the range of hundreds or even thousands of square meter per gram (e.g., the highest experimental BET surface areas on the order of 7000 m^2^ g^−1^ have been reported for metal–organic framework materials [95]), contrary to macroporous (pore size greater than 50 nm) or non-porous substrates with specific surface areas rarely exceeding 100 m^2^ g^−1^.

High binding affinity and selectivity recommended according to the next two criteria are achieved essentially in three different ways: (i) careful matching of the chemical character between the adsorbate and the adsorbent surface (e.g., hydrophilic surfaces to adsorb water vapour), (ii) the use of solid substrates possessing uniform pores capable of confining selectively the main component of the gas mixture on the basis of differences in molecular size, (iii) surface modification of the existing supports by physical or chemical deposition of appropriate functionalities that will provide active sites for electrostatic or chemical binding of polar adsorbates [91,96,97,98,99]. For materials possessing charged surfaces, the substitution of the original charge-compensating ions by higher valence counter-ions has been also tested for energy storage by adsorption of polar vapours [100]. With small-pore supports, special attention should be paid to the functionalization procedures in order to prevent the modification of pore accessibility or even blocking the entrance of some pores [91].

Nevertheless, the main drawback of porous solids, and the microporous ones in particular, is their great sensitivity to deactivation by insufficient regeneration, which may compromise the reversibility of the charge-discharge cycle upon energy storage. It is worthwhile noting here that the insufficient regeneration usually leaves the adsorbate species held most strongly on the adsorbent surface, i.e., the molecules which have been retained on adsorption sites characterized by the maximum energy of adsorption. Enhanced adsorption affinity and selectivity of the adsorbent towards the adsorbate molecules may pose similar regeneration issues, even though the adsorbent is non-porous or macroporous. In all such cases, harsh desorption conditions are usually necessary upon the charging phase to restore the maximum energy during the discharging step. High regeneration temperatures, in turn, can affect the structural integrity of the adsorbent and contribute to the complexity of the heat exchange processes and devices. 

In addition, it is a technical challenge to keep the degassed adsorbent away from the adsorbate from the end of the charging phase until the subsequent adsorption step (i.e., maintain the perfect vacuum in closed sorption systems or keep the trapped air perfectly dry in open sorption systems). The use of adsorbents able to adsorb great amounts of adsorbate at low vapour pressures seems rather prohibitive.

It should also be remembered that the bulk and surface diffusion of the adsorbate in porous media often has a negative impact on the adsorption and desorption kinetics [101,102]. Size exclusion effect may even prevent the active surface sites located within very small pores from being accessed by large adsorbate molecules. In consequence, mesoporous materials seem to be the best compromise between high surface area and fast mass transport requirements. A good strategy to manage such kinetics problems lies in developing materials with tailored hierarchical porosity [103].

The use of powdered materials may have the undesirable consequence of increasing the flow resistance, especially in the particular case of open sorption systems. Solid adsorbents in a pre-shaped, monolithic or granular form are recommended to ensure good heat transfer between air flow and adsorbent surface or to reduce the pressure drop across the packed bed of adsorbent [77]. Special attention should be paid to materials up-scaling and shaping procedures when elaborating a strategy for large-scale production of materials with controlled properties [104,105,106]. In practice, the adsorption performance of pre-shaped materials up-scaled from research to industrial production need not always be as high as that evaluated for small powdered samples under idealized laboratory conditions. As an example, the synthesis up-scaling in continuous mode allows avoiding reproducibility and homogeneity issues that may arise due to batch synthesis [104]. Whenever possible, industrial manufacturing processes of powders compaction not requiring a massive addition of fillers and binders to create macroscopic objects of the desired shape and size are preferred because the filler and binder phases often significantly impact the dimensional change, mechanical, and thermal properties of adsorbents, or even can alter their surface properties (e.g., through pore-blocking effects) [107,108]. Direct synthesis routes to produce monolithic materials with hierarchical porosity have been also developed in parallel [109,110].

## 5. Presentation of Adsorbent Candidates for Adsorption-Based Thermochemical Storage of Energy

In this section, the most pertinent and relevant information is given about the structure and surface properties of potential candidates for adsorbents reported in the literature. In accordance with the arguments put forward in the previous sections, there appears to be much potential for thermochemical storage of energy related with the use of water vapour as the adsorbate. Therefore, the main emphasis here is placed on the type of surface interactions of the selected groups of materials with water molecules in the gas phase, as well as on their structural and textural integrity under hydrothermal conditions. There is also an overview of the principal synthetic routes to obtain target adsorbents or the surface modification procedures to enhance the adsorption properties of the natural solid supports. The more technical details related to their practical use, as considered by research teams in the framework of R&D projects, or inferred from laboratory-scale pilot testing or industrial practice, are presented whenever they have been published in the corresponding articles.

To enable feature comparison, various adsorbent groups are reported in Table 3 together with specific surface area, type of porosity, as well as characteristic adsorption capacity and heat data.

**Figure 3:**
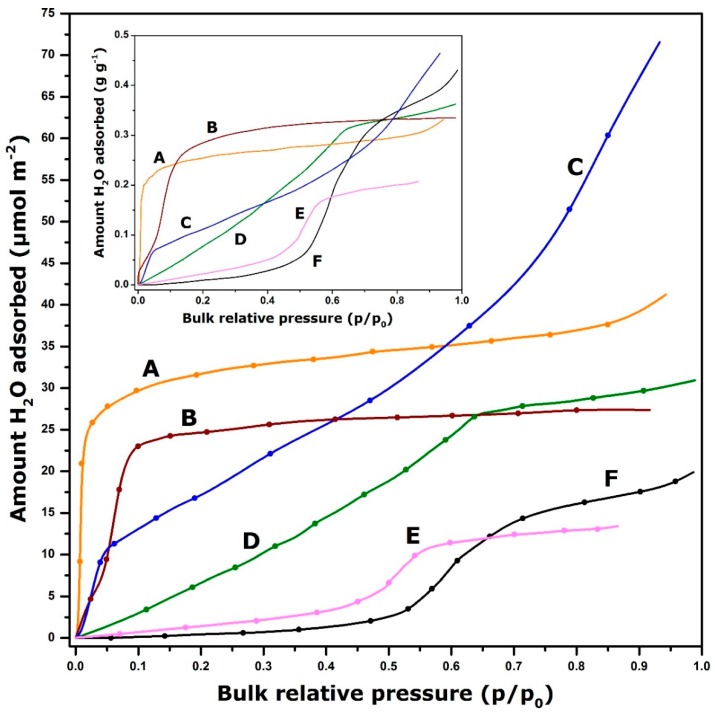
Representative shapes of equilibrium adsorption isotherms for water vapour onto different adsorbents at 298 K (with the exception of silica gel and ionosilica: data at 301 K and 313 K, respectively): zeolite 13X (Curve A); silicoaluminophosphate molecular sieve SAPO-34 (Curve B); activated alumina F-200 (Curve C); silica gel, Mobile Sorbead R (Curve D); ionosilica A-Cl (Curve E); activated carbon BPL (Curve F). The amount of H_2_O adsorbed expressed in µmols of adsorbate per unit surface area of the adsorbent. The inset shows the same curves on a different ordinate scale: the quantity of adsorption expressed as mass of the adsorbate per unit mass of the adsorbent. Data adapted from [119,120,121,122,123,124].

As has been argued before, the storage performance of a given working couple under specific conditions will depend on the mechanism by which water vapour is retained on the solid surface. This mechanism is usually reflected in the particular shape of the adsorption isotherm. Figure 3 shows some representative shapes of adsorption curves for selected adsorbents. 

By analogy with the IUPAC classification of experimental adsorption isotherms for gaseous nitrogen at the temperature of liquid nitrogen (77 K) [52,125], zeolite 13X exhibits an isotherm similar to the Type I curve possessing a very steep initial portion and a limiting water uptake at higher relative pressures of water vapour (curve A). These features reflect a high affinity of water vapour towards the solid surface even in the low pressure region. As a consequence, a much deeper regeneration of the adsorbent is needed upon drying (i.e., desorption) to remove all water molecules from the surface by going down to near zero relative pressure or/and by heating up to high temperatures so as to restore the maximum energy during the discharging step (i.e., adsorption). The Type II isotherm has been obtained with activated alumina (curve C). Here there is still a marked affinity between the adsorbate and the adsorbent and the adsorption phenomenon likely follows unlimited monolayer-multilayer mechanism. In the case of activated carbon (curve F), the adsorption isotherm is convex to the axis of relative pressures up to a p/p_0_ about 0.5. This points out the weak interaction of water molecules with a carbonaceous surface, despite the enhancement of the heat of adsorption within the micropores [126]. Further increase in the water uptake in the intermediate pressure region gives rise to the so-called *S-shaped adsorption curve* with the limiting water uptake over a range of high p/p_0_ values. An adsorption isotherm of the same shape has been obtained when adsorbing water vapour onto ionosilica (curve E); the point of inflexion is shifted towards lower p/p_0_ values. One advantage of such adsorption systems is that it may not be necessary to apply an effective deep vacuum evacuation or a high temperature treatment during the charging step, thereby making the adsorbent regeneration to proceed under milder conditions. For adsorbents giving stepped adsorption isotherms with a hysteresis between the adsorption and desorption branches (e.g., Type V isotherms), the lower is the relative pressure corresponding to the desorption step, the higher the regeneration temperature will be. Curves D and B are an intermediate between Types I and S-shaped isotherms in line with the more or less diminished adsorbent-adsorbate affinity, which manifests itself by a convex shape in the beginning of the adsorption range.

Nonetheless, it should be still remembered that the amount of water vapour retained on the surface, *on a per unit surface area basis*, between the charging and discharging steps is not the only condition to yield high energy gains. Certainly, it is strictly related to the surface density of reactive sites responsible for water adsorption. However, a higher specific surface area often compensates for lower density of surface sites, as is illustrated in the inset graph in Figure 3. Furthermore, the knowledge about the differential heat of water adsorption and its variations with the surface coverage ratio is crucial for the selection of suitable adsorbents.

### 5.1. Amorphous Silicas and Aluminoslicates

This large class of materials includes silica gels, aerogels, precipitated silica powders, pyrogenic silica, ordered mesoporous silicas and aluminosilicates, silicas and aluminosilicates with hierarchical porosity, silica nanoparticles with different pores, morphologies and sizes, periodic mesoporous organosilicas [127,128,129,130,131,132,133].

Pure silica gels are generally obtained by sol-gel processing in aqueous solutions where repeated hydrolysis of silane and condensation of the siloxane bonds result in aqueous polysilicate species evolving under appropriate conditions into essentially anhydrous SiO_2_ in monolithic or powdered form. Silica aerogels having a sponge-like porous structure are produced by drying the aged silica gels under conditions necessary to prevent the collapse of the gel structure. Precipitated silica is manufactured by precipitation of sodium silicate by sulfuric acid under aqueous alkaline conditions, while stirring at elevated temperatures to avoid the formation of a gel. Pyrogenic or fumed silica is usually prepared by the hydrolysis of chlorosilane in a flame of hydrogen and oxygen at high temperatures (1273 K or higher). Although the density of pure amorphous silica is about 2200 kg m^−3^, silica gels, aerogels or pyrogenic silica have much lower bulk densities because of their porosity. Monodispersed silica particles may be also synthesized by the so-called Stöber method in which the hydrolysis of silicon alkoxides occurs in a mixture of alcohol and water when making use of a catalyst. Such silica structures represent 3D networks of silicon-oxygen tetrahedra inter-connected via bridging oxygen atoms. If all tetrahedra possess silicon centres, the whole framework is electrically neutral.

Since the publication of the surfactant-assisted synthesis of periodic mesoporous silica of the MCM-41 type by the researchers of Mobil Research and Development Corporation [134,135], a great deal of effort has been devoted to the preparation of nanostructured oxide materials with tuneable pore sizes and shapes. With the use of supramolecular surfactant or polymer aggregates to template and direct the formation of porous structure based on either the Cooperative Self-Assembly or True Liquid Crystal Templating mechanism, it was possible to ensure, to the maximum extent possible, the control of not only the pore size, shape, and structural hierarchy, but also the size, shape and morphology of particles. Typical members of this category of silicate materials prepared by ionic and neutral surfactant templating possess framework-confined uniform pores with a tuneable size located near the lower end of the mesopore range and amorphous pore walls of a given thickness; they have large specific surface areas in the order of 1000 m^2^ g^−1^ and their porosity may be as high as 80% of the total volume [136,137,138]. The silica precursor (e.g., fumed or colloidal silica, tetramethyl- or tetraethylorthosilicate-TMOS or TEOS), surfactant template, auxiliary compounds (e.g., 1,3,5-trimethylbenzene, TMB), and reaction conditions (e.g., type of solvent, temperature, aging time, reactant mole ratio, and pH of the reaction medium) can be adjusted to control the pore architecture. Periodic Mesoporous Organosilicas (PMOs) were also developed on a similar basis by the combination of an appropriate surfactant template and a silsesquioxane as the organosilica precursor.

Depending on the chemical character and spatial distribution of functional groups localized on the surface after the synthesis pathway and post-synthesis treatments applied, the above solids exhibit surfaces with more or less pronounced hydrophobic-hydrophilic behaviour when interacting with water in a gaseous state and other polar vapours. The surface chemistry of silica materials may also be modified by a post-synthesis grafting of metal centres or organic functional groups due to the presence of hydroxyl groups that are chemically bonded to silicon atoms on the surface (i.e., external or surface silanol groups) which may act as anchoring sites for metal species or silane/silazane coupling agents for organic moieties [96,130,131,139]). It is also worth noting here that hydrothermal and mechanical stability of the porous structure in silica-based materials are usually regarded as closely related to surface hydrophobicity [131]. 

There exists an interesting way to create surface acidity on silica. Certain bases (e.g., ammonia, pyridine, or 2,6-dimethylpyridine) initially H-bonded to silanol groups on the silica surface may be protonated upon the addition of gaseous SO_3_ or NO_2_ [140]. It appears that sufficiently strong Lewis acids can interact directly with the oxygen atom of a silanol group (i.e., nucleophilic attack on the acid by the lone pairs on oxygen). Chemisorption of SO_3_ or NO_2_ molecules followed by a proton transfer from the silanol to the oxygen atom of the covalently bonded acid group leads to the formation of S−OH or N−OH functionalities with induced Brønsted acidity. Alternatively, a great variety of materials with enhanced surface acidity or basicity (e.g., amino, carboxylate or dihydroimidazole surface groups) for capture of basic or acidic gases may potentially be prepared through chemical modification with functionalized alkoxysilanes directly during the synthesis (i.e., co-hydrolysis with the appropriate silica precursor). In the case of aluminosilicates, the substitution of silicon atoms in their tetrahedral positions by aluminium ones, via one-pot direct synthesis using an adequate mixture of precursors, creates a negative charge which is neutralised mostly by protons or alkaline cations. It should be, however, noted that the degree of such an isomorphous substitution strongly depends on the preparation method and the aluminium precursor, frequently resulting in the appearance of extra-framework species (e.g., octahedrally co-ordinated aluminium) [130].

As a final remark, it is important to highlight here that the use of hybrid organic-siliceous materials for heat storage should be generally excluded not only when the surface reactivity towards water vapour is reduced to a considerable extent (e.g., hydrophobized surfaces), but also when the thermal degradability of organic moieties exposed on the silica surface could compromise the stability of repeated charging-discharging cycles.

#### 5.1.1. Surface Reactivity and Hydrothermal Stability of Amorphous Silica Materials

The *surface reactivity* of silica is mainly due to the presence of *silanol groups* (≡Si−OH), which make such a surface hydrophilic, and *siloxane groups* (≡Si−O−Si≡), which impart more hydrophobic character to the surface because of the back bonding of oxygen lone pair electrons into d-orbitals of silicon. The permanent dipole moment of a silanol group is responsible for physical adsorption via dipole-dipole or dispersion interactions of the silica surface with polar molecules or aromatic hydrocarbons at the Solid-Gas interface. A rather weak acidity of silica surface, characterized by irreversible adsorption (i.e., *chemisorption*) of such basic molecules as pyridine or ammonia, is mostly due to the hydrogen-bonding propensity of silanols; indeed, their proton transfer power was evaluated, by analogy with the acidity in aqueous solution, as corresponding to the apparent pK_a_ value being close to 7 [141]. Amongst various types of silanol groups that may be found on a silica surface, three types are believed to play a key role in determining the surface behaviour of silica materials: *isolated*, *geminal*, i.e., silanediols, $=Si{\left(OH\right)}_{2}$, and *vicinal*, i.e., adjacent OH groups hydrogen bonded to each other.

When interacting with water molecules adsorbed from the gas phase, the isolated and vicinal silanols can potentially act as hydrogen-bond acceptors and donors, thereby being involved in two H-bonds with two water molecules; the geminal OH groups likely form the hydrogen bonded ring structures. Based on ab initio molecular dynamics simulations at room temperature, Cimas et al. have also demonstrated that the interfacial water layer on amorphous silica was on average much more disordered and less mobile than that formed on a crystalline quartz surface [142]. As a corollary to these hypotheses, the zero-coverage heat of adsorption, corresponding to the exothermic adsorption of the first water molecules on a hydroxylated surface, was found to be about 60 and 80 kJ mol^−1^ for amorphous and crystalline silica, respectively [31]. When only one H-bond is formed per one adsorbate molecule on isolated silanols (e.g., on dehydroxylated silica), the corresponding heat of adsorption falls below 44 kJ mol^−1^ and hardly depends on the surface coverage ratio. For gaseous NH_3_ molecules adsorbed onto amorphous silica, the zero-coverage heat of adsorption is about 80 kJ mol^−1^ on hydroxylated silica and it passes to about 60 kJ mol^−1^ after preliminary dehydroxylation [31].

The total density of the above mentioned surface functionalities, their mutual proportions, as well as their reactivity depend on the surface hydration state and the local surface topology, thereby being sensitive to the preparation method, thermal treatment applied, and porosity of the material [142,143,144,145,146,147]. The surface density of silanols on a completely hydroxylated amorphous silica has been a subject of controversy in the scientific literature; it should, however, be remembered that various data reported in numerous publications were obtained based on different experimental techniques and data processing methods. For example, considered as a physicochemical constant according to the Zhuravlev model (about 5 OH nm^−2^ [143,148]), the overall density of silanols attached to the surface of silica gels was found to range between 3.8 OH nm^−2^ and 6.7 OH nm^−2^ depending on the mechanism of silanol condensation during surface activation by heating (data inferred from thermogravimetric analysis) [149]. The vicinal silanols likely predominate on silica materials with fine pores and their concentration decreases with increasing pore size to the extent that they are practically absent from large-pore silica samples [144].

Dehydroxylation of a silica surface by vacuum or flow-degas treatment, or/and by heating at higher temperatures induces a progressive loss of silanols, which are converted to siloxane groups on the surface. The vicinal groups begin to condense at temperatures about 473–573 K, whereas the condensation of isolated silanols requires much higher temperatures (e.g., 973–1073 K) as a consequence of their limited mobility [147,150]. Despite some controversy about the reversibility of silica dehydroxylation, the siloxane-to-silanol interconversion during hydration-dehydration of amorphous silica is admitted to remain completely reversible at least until 673 K. The plausible explanation is that dehydration produces strained siloxane bonds belonging to multi-membered siloxane rings which are very reactive and may be thus easily re-opened upon exposure to water vapour to form more stable silanol-containing structures; high temperature-induced reorientation of silicon-oxygen tetrahedra to relieve the strain generated at the surface decreases the siloxane susceptibility to rehydration. Nevertheless, boiling of the dehydroxylated silica surface in water can restore completely or partially the maximum surface coverage with silanol groups, even though the thermal pre-treatment has been carried out at temperatures close to 1273 K [143,146].

In consequence, silica surfaces calcined or prepared at high temperatures possess substantially greater relative populations of siloxanes. For example, the surface of a pyrogenic silica is rich in siloxane groups and it has a predominantly hydrophobic character. The breaking of siloxane bonds via dissociative chemisorption of water vapour leading to the formation of surface silanols is still possible [151]. On the contrary, non-calcined samples or those exposed to humid environment, like silica gels, aerogels, and precipitated silica, exhibit hydrophilic surface properties which may diminish when the activation temperature is raised [149].

#### 5.1.2. Surface Reactivity and Hydrothermal Stability of Ordered Mesoporous Silicas and Alumino-silicates

In the case of amorphous aluminosilicates achieved by the isomorphous substitution of silicon by aluminium, clear evidence has been provided for the existence of strong Brønsted acid sites which take the form of hydroxyl groups located between aluminium and silicon atoms occupying the adjacent tetrahedra, i.e., the so-called “*bridging*” *hydroxyls*, ≡Si−O(H)−Al≡ [141,152,153,154]. The most favourable sites for substitution are those that result in bridging structures involving a silicon atom bonded to three adjacent SiO_4_ tetrahedra (i.e., Q_3_ silicon atom according to the NMR formalism) [154]. Increasing aluminium content in the silica framework causes an increase in the surface density of bridging hydroxyls, thereby enhancing the surface acidity character. For example, the heat of NH_3_ adsorption onto proton-exchanged aluminosilicate of the MCM-41 type at very low surface coverage ratios was measured to be about 150 kJ mol^−1^ [155]. In line with the well-known empirical observation referred to as Loewenstein’s rule [156], the maximum substitution of the silicon in 3D frameworks and plane networks of tetrahedra by aluminium should not exceed 50% (i.e., the existence of ≡Al−O−Al≡ bridges is to be avoided). The relationship between the surface density of bridging hydroxyls and the silicon-to-aluminium ratio is rarely linear because of the formation of extra-framework Lewis species, as a function of the synthesis pathway, aluminium precursor, or post-synthesis thermal treatment. The surface hydroxyl density of ordered mesoporous silicas and aluminosilicates is usually in the range of 2–4 OH nm^−2^ [155,157]. It is worthwhile noting that the surface energy characteristics of ordered mesoporous silicas and aluminosilicates are significantly smaller than those of typical hydrophilic silicas [155]. Finally, the preparation of silica-based composites via deposition of inorganic hygroscopic salts on the silica or aluminosilicate surface by incipient wetness impregnation should also be mentioned in the context of the present review [68].

The thermal and hydrothermal stability of ordered mesoporous silicas and aluminosilicates is usually lower than that of typical amorphous silica materials. For example, the pore structure of the MCM-41 silica was found to collapse at calcination temperatures above 1073 K [158]; the incorporation of Al in the framework of MCM-41 showed little effect on its hydrothermal stability in an air stream containing 3–20 vol% water vapour at 873 K [159]. The degradation mechanism for pure mesoporous silica is mainly related to the hydrolysis of Si−O−Si bonds exposed to water vapour; it begins on the outer regions of the pore walls and propagates inwards [159,160]. Therefore, it is important to protect the exposed silica surface to improve the hydrothermal stability of the porous material. Among the different protection techniques available, the deposition of aluminium onto the silica framework is considered as being quite efficient, especially in the case of materials characterized by thicker pore walls [160,161]. Nevertheless, it should not be forgotten that, when significant amount of aluminium is introduced into the silica framework, the dealumination process upon activation or calcination at high temperatures not only compromises the hydrothermal stability, but it also results in a decrease in the Brønsted-type acidity of the sample, thus enhancing the Lewis-type acidity [153].

#### 5.1.3. Adsorbents for Heat Storage by Gas-Solid Adsorption

Silica gels, like commercial type A and RD samples produced by Fuji Davison, exhibited a significant adsorption capacity towards water vapour with a maximum uptake being in the range of 0.4–0.45 g of water per gram of the solid sample. The related isosteric heat of adsorption was reported to be around 2.7 kJ g^−1^ under the typical operating conditions of adsorption chillers, namely the temperature and pressure ranging, respectively, between 298 and 338 K and between 500 and 7000 Pa [162]. The temperatures necessary for adsorbent regeneration were relatively low. For example, Ng et al. compared the regeneration and adsorption characteristics of Fuji Davison type A, 3A and RD silica gels, and concluded that desorption at 363 K was sufficient to recover 95% of the initial adsorption performance in all cases [163]. Despite the good theoretical adsorption performance and easy regeneration, the main drawback of silica gels was considered to be related to the weak hydrophilic character of their surface within the particular working window including the desorption step carried out at 423 K and 5.6 kPa and the adsorption one performed at 308 K and 1.2 kPa, especially for closed storage systems [87]. 

Ito et al. synthesized a silica gel by adding aluminium ions as a silica growth inhibitor which resulted in a sample having a pore diameter reduced by about 10%; the water vapour adsorption by the sample at low relative pressures and its stability after 100 repeated adsorption-desorption cycles were shown to be improved [164]. Knez and Novak prepared a silica aerogel by supercritical CO_2_ drying, thus achieving a significantly higher adsorption capacity towards water vapour in the range between 1 and 1.2 g g^−1^ mainly due to its much higher porosity up to 99%. Nevertheless, the adsorption capacity was found to decrease markedly after the first cycle owing to the collapse of the porous structure during the dehydration step. When physically mixing silica with alumina aerogels, it was possible to increase the number of stable cycles up to 25 [111].

In order to cover both the need for hot water and space heating in a single-family house, the Modular High Energy Density Sorption Heat Storage (MODESTORE) research project tested the use of the silica gel-water vapour working pair in a closed system [165,166]. The prototype was developed on the basis of 200 kg of silica gel contained in a cylindrically shaped reactor equipped with a heat-exchanger pipe arranged inside in a serpentine. Several shortcomings in the system, namely the temperature loss on account of the low heat conductivity of silica gel, some important losses in the sensible heat of liquid water after the charging step, or the need for additional heat of evaporation before the discharging step, reduced the efficiency of the prototype [165]. In 2005, a pilot using a MODESTORE reactor and a storage tank containing 1000 kg of silica gel was installed in Austria. Contrary to the expectation of a good performance, the energy density reached only 140 MJ m^−3^ (i.e., 39 kWh m^−3^) much below the theoretical value of 684 MJ m^−3^ (i.e., 190 kWh m^−3^) [166].

A new family of composite materials called *selective water sorbents* (SWS) was proposed in view of water sorption uses. Their synthesis was based on the concept of tailoring of the host porous matrix at nanometer level by adding an inorganic salt inside the pores (e.g., “a salt in a nanoporous matrix” composites) [167]. Such hygroscopic salts as Ca(NO_3_)_2_, CaCl_2_, LiNO_3_, LiBr, MgCl_2_, NaSO_4_ or CuSO_4_ were introduced into micro- or meso-porous silica gels or aluminosilicates in order to enhance the surface hydrophilic character, thus improving both the water sorption capacity and the heat evolved during the discharging phase [91,114,115,167,168]. Nevertheless, the formation of saline solutions within the pores under certain hydration states was observed with a potential consequence of salt leakage leading to the reduced cyclability and thus requiring a proper organization of the process [169].

Ordered mesoporous silicates and aluminosilicates of the MCM-41 and SBA-15 type were also considered as efficient adsorbents for water vapour and the adsorption mechanisms, as well as the modification of their surface or textural properties, were studied by several research teams [92,170,171,172,173]. Llewellyn et al. argued that water molecules were initially adsorbed on hydroxyl groups present at a relatively hydrophobic surface of MCM-41 samples; this initial step was followed, when progressively increasing the equilibrium pressure of water vapour, by the formation of clusters and capillary condensation, therefore leading to a total filling of the pore volume and resulting in a type V isotherm according to the IUPAC classification [170]. Heating up to 500 K under vacuum conditions caused the loss of physisorbed water; above this temperature, the loss of chemisorbed water and surface dehydroxylation were observed. Kocherbitov and Alfredsson revealed two driving forces balancing the mass of condensed water and the surface area covered in the process of capillary condensation: saturation of hydrogen bonds of pre-adsorbed water molecules and condensation of water at low relative humidity values [171]. Kittaka et al. [172] demonstrated that adsorption-desorption hysteresis loops observed in the case of MCM-41 and SBA-15 samples shifted to higher pressures with an increase in the pore size, as for typical mesoporous materials. Rozwadowski et al. showed that the increasing Al content in the framework of MCM-41 aluminosilicates caused a decrease in the BET surface area and an unexpected reduction in the sorption capacity towards water vapour [173]. These undesirable effects were explained by the formation of clusters of liquid water around the hydrophilic Al centres which resulted in clogging the pores. According to Jabbari et al. [92], the presence of an aluminium salt finely dispersed inside the pores of the substrate made the water molecules to be attracted within the pores which facilitated the hydration of both the salt and the supporting material. It is worth noting that various Al-MCM-41 and Al-SBA-15 composites exhibited much higher sorption capacities (i.e., up to 0.17 and 0.09 g of water vapour per gram of the sample, respectively) at a relative pressure of about 0.3 than those observed for pure host materials (0.04 and 0.02 g g^−1^ for MCM-41 and SBA-15, respectively). More recently, Thach et al. reported highly hydrophilic ionosilicas, known as mesoporous silica-based hybrid materials containing covalently bound ionic groups [124,174]. The hydrophilic character of such solids was shown to originate from both the silica network and the high number of immobilized ionic ammonium species. The preparation of the ammonium substructure of the precursor combined with an exchangeable counter-ion allowed not only to adjust this hydrophilic character but also to enhance cycling stability upon water adsorption. 

### 5.2. Zeolitic Materials

Zeolites represent a broad family of microporous, aluminosilicate minerals that occur naturally and are also synthesized in the laboratory or produced industrially on a large scale. The crystalline aluminosilicate framework is built of [TO_4_] tetrahedra inter-connected via bridging oxygen atoms, where T is either a tetravalent silicon atom or a trivalent aluminium one [157,175]. Like in the case of amorphous aluminosilicates, the formation of the three-dimensional structure of tetrahedra is consistent with the Loewenstein’s rule [156]. Nevertheless, it is worthwhile to mention here that the first case of violations of the Loewenstein’s rule in high and low silica (LS) zeolites in the protonated form has been recently predicted when screening the aluminium distribution on the basis of density functional theory [176]. The primary building units are assembled into some more complex structures called Secondary Building Units (SBU), like octahedra, cubes, truncated octahedra (also named *sodalite cages*) or prisms with a square or hexagonal base, which form a regular porous framework with cavities (or cages) and channels possessing calibrated dimensions [170]. Since the presence of [AlO_4_]^−^ tetrahedra makes the zeolite framework negatively charged, this negative charge is compensated by the adsorption of protons or other cations at the extra-framework positions. In the case of industrially produced zeolites, such *compensating cations* often include sodium Na^+^ ions due to its importance in industrial processes [177]. Other cations of alkali or alkaline earth metals may also assure electroneutrality of the zeolite structure [178]. The general chemical formula of zeolites can be presented in the following form: $M_{\frac{x}{n}}[{\left(AlO_{2}\right)}_{x}\left(SiO_{2}{)}_{y}\right]\cdot mH_{2}$, where M and n stand for the compensating cation and its valence, respectively; x and y denote the numbers of [AlO_4_]^−^ and [SiO_4_] tetrahedra, whereas m corresponds to the number of residual water molecules in the natural state. 

Hydrothermal synthesis is the most commonly used procedure to prepare synthetic zeolites [157]. Typical synthesis carried out in batch reactors includes several steps. In the first step, solutions of sodium silicate, sodium aluminate, and sodium hydroxide (synthesis under basic conditions) are mixed together which leads to the formation of a gel in line with the condensation–polymerization pathway during which Si-O-Si and Si-O-Al chains are created. The zeolite structure is crystallized through a nucleation step, followed by a crystal growth involving assimilation of aluminosilicate from the solution phase in a closed hydrothermal system (an autoclave under autogenous pressures) at temperatures usually between 298 and 448 K [179]. The time for zeolite crystallization ranges from a few hours to several days [180]. By varying reaction conditions (e.g., temperature, time, and degree of agitation) or nature of the structuring agent, it is possible to create a large number of crystalline aluminosilicates with different pore sizes and silicon-to-aluminium ratios (i.e., Si:Al ratio) in the final material. It has been also demonstrated that the continuous flow synthesis of ZSM-5 may greatly accelerate the crystallization from amorphous state to full crystallinity; namely, it can completed in the tens of seconds range or even within several seconds [181].

Cation exchange with alkali, alkaline-earth, and transition metals or lanthanides is an important method often used to modify the nature and number of compensating cations, thereby tuning the surface properties of zeolites [157,175,178,182,183,184]. The propensity of these materials to adsorb various adsorbates, and in particular water vapour, may be easily altered in such a way [100,178]. 

#### 5.2.1. Surface Reactivity and Hydrothermal Stability of Zeolites

Microporous structure of zeolites imparts these crystalline materials with high specific surface areas (e.g., about 800 m^2^ g^−1^ for zeolite 13X [185]), which enhances their capacity to adsorb great quantities of such gaseous molecules as H_2_O and CO_2_. The adsorption isotherms for water vapour are of type I according to the IUPAC classification, and they usually do not exhibit any hysteresis loop between the adsorption and desorption branches [186].

Sorption performance and hydrothermal stability of zeolites depend on whether they are present in the protonated form or in a metal cation-containing one [187,188,189,190]. The compensating metal cations can be located within different Secondary Building Units or at regular polygon windows ensuring communication between such units [178]. They are not chemically bonded to the zeolite framework but they occupy various crystallographic positions characterized by different potential energies. Three physical factors appear crucial for cation location: (i) coordination with oxygens of the zeolite framework, (ii) cation-cation repulsions, and (iii) coordination with negatively charged moieties of the adsorbate species. Two types of zeolite structures are of interest for industrial uses in adsorption, namely types A zeolites (Linde Type A or LTA) and faujasites (FAU) of type X or Y. 

**Figure 4:**
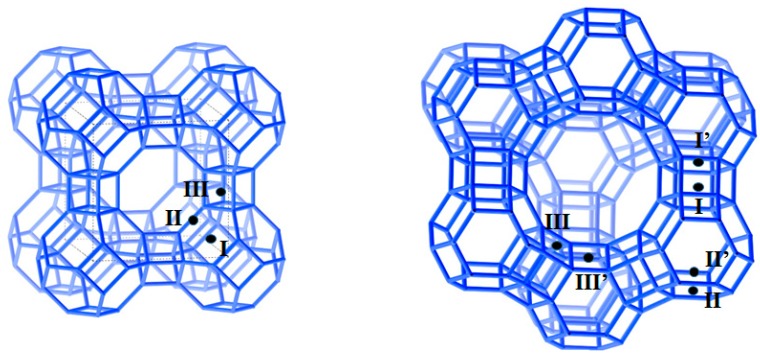
LTA (left) and FAU (right) zeolite structures showing the principal crystallographic sites for the location of charge-compensating cations. Made on the basis of the two zeolite structures extracted from database [191].

The LTA framework consists of sodalite units which are connected by their square faces through square prisms. The unit cell is cubic with $Fm\bar{3}c$ symmetry. Eight sodalite cages arranged in a cubic structure surround the so-called *supercage* with a minimum free diameter of 1.14 nm, which constitutes the primary type of pore in the zeolite (c.f., Figure 4). These pores are disposed perpendicular to each other in the x, y, and z planes and form a 3-dimensional pore structure. The access to supercages is ensured through eight-member oxygen-rings (windows) with a minimum diameter of 0.44 nm. The size of the windows depends also on the number and nature of charge-compensating cations [157,177]. Figure 4 shows the most probable locations for the compensating cations in the LTA zeolite: (i) site I, at the centre of a six-member oxygen ring constituting one of the eight corners of the supercage; (ii) site II, close to the eight-member oxygen window thus directly obstructing the entrance to the supercage; (iii) site III, close to the four-member oxygen ring inside the cavity [157].

In the FAU framework, the sodalite units are linked through hexagonal prisms and 10 truncated octahedra surround a supercage (the minimum free diameter is equal to 1.25 nm), to which the access is through 12-member oxygen rings (windows) with a free diameter of about 0.74 nm [157]. The unit cell is also cubic with $Fd\bar{3}m$ symmetry. The FAU framework is characterized by the greatest supercage volume amongst all known zeolites and its total void fraction is about 50%. Types X and Y zeolites have the same skeletal structure; the only difference is that the formers contain between 96 to 77 aluminium tetrahedra of the total 192 tetrahedra in the unit cell (i.e., Si:Al ratio varies from 1 to 1.5) and the latters contain between 76 and 48 [AlO_4_]^−^ tetrahedra (i.e., Si:Al ratio changes from 1.5 to 3).

The principal crystallographic sites for the location of charge-compensating cations are shown in Figure 4. They include: (i) site I, in the hexagonal prism, either in its centre or shifted from it; (ii) site I’, in the sodalite cage toward the hexagonal prism (i.e., close to the 6-ring window); (iii) site II, in the supercage, at the centre of the 6-ring window between the sodalite cage and the supercage; (iv) site II’, in the sodalite cage close to the 6-ring window; (v) sites III and III’, in the supercage, close to its 12-ring window [178]. In the case of Y type faujasites containing monovalent compensating cations, the increase in the cation size favours the occupation of the most confined sites I, whereas for divalent cations sites I’ are the preferred positions [178,183] It is important to realize here that hydration of zeolites will affect the location of compensating cations, thus leading to cation redistribution upon water adsorption as a function of the hydration extent [100,178,192,193]. For example, Di Lella et al. [192] reported cation redistribution upon water adsorption for sodium faujasite Y (i.e., NaY) with varying cation contents (Si:Al ratio = 1.53–3). For a dehydrated sample containing initially less than 16 Na^+^ in site I, cation migration towards site I’ was postulated as a consequence of too small space being offered for hydrated cations within the hexagonal prism. For NaX zeolites with low Si:Al ratio [193], numerous extra-framework Na^+^ cations were supposed to be mainly located in sites I’ and II, and also occupy sites III’ at the dry state; the sodium distribution appeared hardly affected upon water adsorption. A complete ion exchange of sodium by calcium in faujasites was shown to be possible and sites I and II were regarded as the preferred centres for calcium location [178]. However, in samples containing a mixture of Na^+^ and Mg^2+^, the two types of compensating cations shared mostly sites I’ and II. The trend for moving towards more confined sites to occupy to a larger extent the sites II close to the sodalite cages was revealed at higher temperatures (≥723 K [100]), irrespective of the cation nature. In the case of hydrated MgNaX and CaX zeolites, Na^+^ cations tended to occupy more sites I and IV (i.e., at the centre of the supercage), and less sites of the type II, whereas divalent analogues were rather located on sites I’, II, and III [100].

Brønsted and Lewis acid sites may be present in different zeolites [45]. The Brønsted acid sites represent a hydrogen atom bonded to an oxygen one to form a surface hydroxyl group, in which the oxygen either belong to a framework aluminium tetrahedron (i.e., terminal –OH) or constitutes a bridge between adjacent silicon and aluminium tetrahedra (i.e., bridging –OH). Such surface acidic sites may be formed in four different ways: (i) by exchanging the pristine compensating cations with ammonium ones followed by a calcination step to eliminate ammonia, (ii) by cation exchange in acidic medium (high silica zeolites), (iii) by breaking an Al–O–Si bond in the zeolite framework, thus resulting in the formation of surface Si–OH and Al–OH groups, and (iv) via hydrolysis of multivalent compensating cations [183]. Lewis acid sites in zeolites represent electron deficient groups which tend to accept electrons when interacting with extra-framework molecules. The following three chemical moieties may exhibit this behaviour: (i) tri-coordinated aluminium atoms exposed at the surface as a consequence of the dehydration of some Brønsted acid sites during calcination or ion exchange (ii) extra-framework aluminium oxide clusters, AlO^+^ or Al_x_O_y_^n+^, produced by delamination process, and (iii) exchangeable compensating cations [194]. In general, decreasing the Si:Al ratio in the zeolite framework causes an increase in the number of compensating cations or protons, thereby enhancing the surface acidic character. Nevertheless, when the pore space becomes crowded with protons, the strength of the related Brønsted acid sites may decrease. For example, in high silica zeolites (Si:Al > 10), the acidic sites are isolated and strong, though they are present in small quantities; on the contrary, in low silica zeolites, these sites are more numerous but weaker.

Synthetic or cation-exchanged X and Y zeolites with a low Si:Al ratio possess only a few protons as charge-compensating species and, therefore, their surface reactivity will be dominated by Lewis-type acidic sites including mostly the extra-framework cations and other electron deficient centres; the presence of bridging hydroxyls groups representing strong acid centres of the Brønsted type is to be rather neglected [100]. Following the molecular dynamics simulation done by Shirono et al. [195], water molecules were shown to adsorb onto NaX (Si:Al = 1) via a three-step mechanism including: (1) adsorption around sodium cations, (2) formation of one molecule-thick adlayer on the pore walls, and (3) pore filling in the supercage during which water molecules were localized around the 12-member oxygen windows. Similar mechanism was postulated for water adsorption onto NaY (Si:Al = 2) with the exception of intermediate hydration states, where the formation of water clusters around Na^+^ cations appeared more plausible than the monolayer adsorption [195].

The adsorption of water vapour onto such protonated zeolites as HY or H-ZSM-5 was argued to occur mainly on Brønsted acid sites, thus resulting in two types of local structures: (i) a neutral complex of two water molecules hydrogen-bonded to a Brønsted acid site and a framework oxygen atom, (ii) an “ion pair” complex formed during simultaneous adsorption of two water molecules by transferring the surface acidic proton to the water dimer [189,190,196,197]. Since the capacity of water vapour adsorption was proven to depend also on the surface topology and the spatial confinement, the exclusive presence of “ion pair” complexes was predicted for HY zeolites by Jungsuttiwong et al. [190].

When accompanied by the migration of charge-compensating cations among various crystallographic sites, hydration and dehydration of zeolites may affect the site accessibility or even block the entrance of some pores. Moreover, marked evolution of the pore architecture of a pre-shaped sample of Na-contained 13X zeolite subject to numerous repeated hydration-dehydration cycles was revealed by Storch et al. [57]. In a general manner, zeolites with a Si:Al ratio ≥ 3.80 appear very stable, whereas those having a Si:Al ratio ≤ 1.28 are quite unstable [58]. Protonated zeolites are usually considered as showing lower thermal stability compared to their sodium-containing analogues, the latter being less stable with increasing framework aluminium [198].

Framework dealumination is the method the most frequently used to enhance the hydrothermal stability of faujasites with higher Si:Al ratios (generally above 2.2). At the industrial scale, zeolite NaY is commonly dealuminated in water steam without collapse of its framework [199]. This can be accompanied by introduction of ammonium ions into the framework and steaming process of NH_4_Y, thus creating extra-framework aluminium at the crystal surface; in consequence H-DAY sample is achieved [200,201]. N. Salman et al. demonstrated that more framework damage by a sour hydrolysis occurred in steam at lower temperatures (473 K), rather than at higher ones (573–973 K) [202]. Nevertheless, the comparison of 3A, 13X and DAY samples in terms of water adsorption capacity and heat of adsorption effect led Kim et al. to conclude that the sorption performance of DAY was weaker than those of the two other samples [185]. Ristic et al. showed that a post-synthesis modification of zeolite Y by a mild HCl treatment and a chemical treatment with H_4_EDTA could diminish (below 413 K) the temperature at which the zeolite should be regenerated to remove all physically adsorbed water [203]. A two-step acid treatment of Mg-exchanged sample could, on the one hand, improve the water sorption capacity, but on the other hand, rise the desorption temperature up to 473 K [203]. Buhl et al. studied the hydrothermal stability of a series of cation-exchanged 13X (Si:Al = 1.18) and LSX (Si:Al = 1.02) zeolites [204]. No framework decomposition was observed up to 473 K for Li^+^, Na^+^, and Ca^2+^ compensating cations, whereas more or less marked framework collapse was detected for K^+^, Rb^+^, Sr^2+^, and Ba^2+^ because of stronger vibrations and repulsive forces exhibited by large cations with increasing temperature, thus destabilizing the framework [204]. Fischer et al. also reported the research work done on the effect of temperature (473–623 K), water vapour pressure, and treatment time on the hydrothermal stability of powdered 13X zeolite and 13X beads [205]. Their results showed that the crystallinity could degrade above 473 K due to the hydrothermal stress, especially under high water vapour pressures. At temperatures in the range of 473–523 K, 13X beads exhibited better hydrothermal stability under lower water vapour pressures.

#### 5.2.2. Zeolitic Adsorbents for Heat Storage by Gas-Solid Adsorption

Though solar energy storage using zeolite-water vapour working couples has drawn much attention since the late twentieth century [16,206], zeolites were intensively studied mainly in view of uses in heterogeneous catalysis. More recently, Gaeini et al. analysed the effect of kinetics and gas flow rate on the thermal performance of a laboratory-scale thermochemical heat storage system, which possessed a fixed-bed reactor filled with zeolite 13X [207]. It was found that slower adsorption process reduced both the efficiency (from around 83% to around 80%) and the power of the reactor (from around 1.4 kW to around 0.6 kW), when the kinetics coefficient was below a threshold value. For each reactor size, an optimum flow rate was established.

Studies of water vapour adsorption onto different cation-exchanged zeolites (Li^+^, Ca^2+^, Mg^2+^) of types X, Y, and A led Jänchen et al. to conclude that the adsorption capacity and integral heat of sorption could be increased as a result of ion exchange with cations of different sizes and charges [69]. Their laboratory-scale storage system based on the use of granulated zeolites yielded storage energy densities up to 810 kJ kg^−1^ (an increase in the storage density of 145%) after the charging step carried out at 453 K. It was also shown later on [208] that a 10–15% higher adsorption capacity and faster kinetics accompanied the storage process performed on binderless zeolite beads of types A and X. The 13X sample appeared as the most promising storage material for charging temperatures up to 470 K. Above this temperature, some degradation of 13X zeolite was observed, especially at high pressures of water vapour, and more stable binderless 4Å zeolite was a better choice in view of potential implementation issues in open and closed storage systems. Herzog et al. attempted to adjust the hydrophilic character of Y-type zeolites by a steaming process and the dealumination was found to lower the desorption temperatures [209]. Gómez-Álvarez et al. concluded, on the basis of molecular simulations, that the nature of the compensating cation affected the adsorption of water vapour to a greater extent than the density of such cations did [210]. It was argued that higher affinity of calcium cations towards water vapour resulted in a more hydrophilic character of the zeolite framework in comparison with that shown by the sodium-exchanged sample, thereby changing the mechanism of surface clustering of water molecules. Li et al. demonstrated that Y-type zeolites finely tailored by ion exchange with Mg^2+^ ions exhibited significantly enhanced vapour uptake capacity, adsorption energy, and intra-crystalline diffusivity compared to the parent samples [211]. Similar conclusions were drawn by Alby et al. for water vapour adsorption of Mg- and Ca-exchanged X-type zeolites under dynamic conditions of gas flow [100]. For example, a Mg-exchanged 13X sample with a cation-exchange ratio of 70% was demonstrated to have much better performance in terms of integral heat release compared to the original Na-X, even though the regeneration conditions were insufficient to remove completely water vapour adsorbed onto zeolite. Stach et al. also showed that Mg^2+^ ions enhanced the water adsorption capacity and integral adsorption heat [212]. They argued that the thermochemical storage performance for various adsorbents depended on the temperatures describing adsorption, desorption, condensation, and evaporation stages.

Some other researchers have tested alternative ways for modification of zeolite performance by salt hydrate encapsulation in a porous structure or by impregnation of the zeolite surface with hygroscopic salts [93,94,117,188]. Cindrella and Dyer compared various zeolites of 4A, X and Y types achieved either by cation exchange with Mg^2+^ and Zn^2+^ ions or by encapsulation of MgCl_2_ and Zn(NO_3_)_2_ salts. Strong interactions were found to occur between the zeolite framework and the salt hydrates, whereas the fibrous structure of the salt-encapsulated zeolite X exhibited an increased surface area for water uptake and promising structural features amenable to thermal energy storage [188]. Whiting et al. tested the use of composite materials obtained on the basis of NaX, mordenite, NaY and HY zeolites impregnated with MgSO_4_ or MgCl_2_ [93,94]. They showed that Na–Y and H–Y samples containing 15 wt% MgSO_4_ produced higher heats upon hydration and dehydration stages (1090 and 876 J g^−1^ of solid, respectively) compared to those of Na–X and MOR composites (731 and 507 J g^−1^ of solid, respectively). Na–Y and H–Y composites containing 15 wt% MgCl_2_ yielded the highest heats of water adsorption (1173 and 970 J g^−1^, respectively), due to the lower deliquescence relative humidity (DRH) of MgCl_2_. Nevertheless, the use of great quantities of MgSO_4_ resulted in significant pore blocking, thus limiting the access of water molecules to the dehydrated salt species. Hongois et al. constructed a storage reactor with 200 g of MgSO_4_-impregnated zeolite 13X [117]. An energy density of 648 J g^−1^ (i.e., 0.18 Wh g^−1^) was achieved during hydration tests and this performance remained unchanged over three charging-discharging cycles, according to adequate micro-calorimetry measurements.

Besides some fundamental research made on open and closed storage systems [77,213,214], several large-scale prototypes have been constructed to test the storage performance under more realistic conditions. Tatsidjodoung et al. built an open prototype operating in the moist-air mode making use of zeolite 13X beads as an adsorbent. With 40 kg of zeolite, an average temperature lift of 38 K was recorded at the outlet of each zeolite vessel for 8 h during the discharging step carried out with an airflow inlet at 293 K, a specific air humidity of 10 g kg^−1^ of dry air, and a flow rate of 180 m^3^ h^−1^ [215]. Johannes et al. designed a STAID prototype composed of two reactors, each containing 40 kg of 13X zeolite [216]. The thermal power output provided about 2.25 kW (namely 27.5 W per kg of the adsorbent) during 6 h [216]. For the purpose of E-Hub project, De Boer et al. realized a storage prototype with 150 kg of 13X zeolite divided in two separate reactors [217]. Unfortunately, the thermal losses due to convection, conduction, and air leakages, as well as the low efficiency of the air-to-air heat recovery unit resulted in a low thermal power output from the system, corresponding only to the 15% efficiency. Gaeini et al. based their 250 L setup on a gas-solid reaction between water vapour and zeolite 13X [206]. The reactor contained four segments of 62.5 L each operating in different modes. A maximum power of around 4 kW was obtained by running the segments in a parallel mode. The Institute for Thermodynamics and Thermal Engineering (ITW) attached to the University of Stuttgart (Germany) was involved in the development of an open storage system called “MONOSORP” [218]. The MONOSORP prototype was developed based on the use of 8 m^3^ of 4A zeolite monolith in the honeycomb structure to improve the adsorption kinetics. With an inlet temperature of about 293 K and a humidity of 6 g kg^−1^ (gram of water per kg of dry air), a maximum temperature lift of around 22 K was achieved. Another open sorption system making use of 13X zeolite as an adsorbent was studied and developed by ZAE Bayern [56]. It was connected to a district heating network in Munich, thus relying on the thermal energy excess produced during night and being independent of the grid during peak demand. The storage density was equal to 446 MJ m^−3^ (i.e., 124 kWh m^−3^) for heating stages, which corresponded to a performance coefficient (COP) value of 0.9.

### 5.3. Other Zeotype Materials

Zeotype aluminophosphates (AlPO) and silico-aluminophosphates (SAPO) possessing frameworks and pore arrangement similar to those of zeolites have attracted much attention in view of adsorbent development for thermal energy storage using water adsorption–desorption cycles. The tetrahedral-framework aluminophosphate (AlPO) was first discovered in the early 1980s by Union Carbide [219]. The zeotype framework was constructed by strictly alternating [AlO_4_]^−^ and [PO_4_]^+^ tetrahedra to obtain an electrically neutral structure. As a consequence of this strict ordering, the framework possessed no odd-membered rings. The surface of such a neutral AlPO framework with no extra-framework cations exhibited more hydrophilic character than that of pure silicate, due to the difference in the electronegativity between aluminium (1.5) and phosphorus (2.1), as well as due to the structural defects in the form of surface P–OH groups [220]. Nevertheless, it still showed less affinity towards water vapour compared to zeolites of type A or type X with anionic aluminosilicate framework and the compensating cations [219].

Because of the moderate hydrophilic behaviour of AlPO framework, the water adsorption isotherms are often of type V, according to the IUPAC classification [221]. This particular behaviour may be advantageous for the heat storage uses: a steep increase in the water uptake within a narrow range of relative vapour pressure argues in favour of low-temperature working conditions, thus decreasing significantly the regeneration temperature of materials down to 363–413 K. [86,222]. However, the total water uptake is somewhat lower when compared with that of faujasites [70]. In order to tune the surface properties, incorporating a fraction of Si atoms into an AlPO framework can produce negatively charged Brønsted acid sites and form a silico-aluminophosphate (SAPO) framework with enhanced hydrophilic character of the material surface [223]. Furthermore, it is also possible to substitute Al and P atoms with some metallic elements to obtain metal-aluminophosphates (i.e., MAlPO), like in the case of FAM-Z01 containing iron cations within the framework. 

Typical synthesis of AlPO or SAPO is carried out at relatively high temperatures (373–523 K) in line with the hydrothermal pathway by making use of structure-directing agents or templates, starting from traditional chemicals containing individual Si, Al, and P atoms or lamellar aluminophosphates afforded from appropriate chemicals. Many parameters may affect the framework formation, such as the source materials, batch composition, pH of the reaction mixture, chemical structure of the template, type of solvent, as well as the crystallization temperature and time [219,224,225,226,227,228].

Two main drawbacks of these materials have limited their massive use as adsorbents in heat storage systems. The first one is related to the framework degradation after a few charging-discharging cycles under hydrothermal conditions [229]. The other one is due to significant preparation costs, especially when expensive templates are to be used (e.g., diethylamine or tetraethylammonium hydroxide) [87,230,231]. Nevertheless, some of these materials have already been commercialized, for example by Mitshubishi Plastics under the name of AQSOA-Z or AQSOA- Functional Adsorbent Material-Zeolite (FAM-Z) [232,233].

Amongst all known AlPO and SAPO materials, AlPO-5, AlPO-18 and SAPO-34 are the most commonly studied samples for thermochemical heat storage uses [60,87,123,167,222,229,232,234,235,236,237,238]. AlPO-5 (or AQSOA-Z05) possesses a particular two-dimensional zeolite-type framework structure having pores with a diameter of 0.74 nm; AlPO-18 has pores with a smaller diameter of 0.38 nm; SAPO-34 (or AQSOA-Z02) framework bears cages with the 0.37 nm windows [239,240].

The mechanism of water vapour adsorption onto AlPO has been investigated by several researchers. Goldfarb et al. used solid state NMR to reveal two types of water molecules retained by AlPO-5: the first type concerned molecules coordinated to the framework Al and the other one represented water molecules physisorbed within the channels of AlPO-5 [241]. Newalkar et al. reported the variations of the isosteric heat of water adsorption onto AlPO-5 as a function of the surface coverage ratio: it was about −88 kJ mol^−1^ at very low coverage ratios and increased sharply to −50 kJ mol^−1^, which was close to the heat of liquefaction of water vapour (−41 kJ mol^−1^). Then it remained almost constant with a further increase in the surface coverage. The authors also suggested that the increase in the water uptake at relative vapour pressures in the 0.2–0.3 range was due to capillary condensation; the initial water uptake occurred within the secondary 6-membered ring channels, and then water molecules filled the primary 12-membered ring channels [220]. Floquet et al. investigated the capillary condensation mechanism by carrying out neutron scattering experiments in the temperature range 280–300 K [236]. For relative vapour pressures below 0.1, the AlPO-5 channels remained empty due to the relative hydrophobic character of the framework. Further increase in the relative pressure up to 0.35 led to the retention of water molecules via interactions only with framework aluminium in octahedral coordination or with defects. Water molecules started to fill the AlPO-5 channels at a relative pressure of 0.35, which was followed by the crystallization of water to form a confined ice-like phase [236]. For AlPO-18 and SAPO-34 samples dried at low temperatures (368 K), Henninger et al. reported water uptake values of 254 g kg^−1^ and 200 g kg^−1^, respectively [86]. They also revealed a steep water retention within a narrow relative pressure range, although the adsorption-desorption isotherm for AlPO-18 contained an undesirable desorption hysteresis. The SAPO-34 samples synthesized by using morpholine as a template were not stable under hydrothermal stress. Changing the template could improve the stability, however this was accompanied by a significant increase in the synthesis cost [86,242]. Goldsworthy studied the adsorption of water vapour onto commercial samples AQSOA-Z02 and AQSOA-Z05 and obtained type IV adsorption isotherms [238]. The maximum water uptake was 0.33kg kg^−1^ for AQSOA-Z02 and 0.235 kg kg^−1^ for AQSOA-Z05. According to Shimooka et al. [232], between two samples FAM-Z05 and FAM-Z02, the former had lower regeneration temperature, though simultaneously lower adsorption capacity. Ristic et al. investigated the influence of the elemental composition and framework structure on the sorption characteristics of AlPO-18, SAPO-34, and a new APO-Tric sample characterized by a triclinically distorted chabazite framework [222]. The heat of adsorption was found to be about 55 kJ mol^−1^ for the three samples, which resulted in an energy density of 864 MJ m^−3^ (i.e., 240 kWh m^−3^) for a regeneration temperature of 413 K. The APO-Tric showed better low-temperature performance and hydrothermal stability, contrary to the gradual water uptake and amorphization of the crystalline structure of more classical SAPO materials after adsorption-desorption cycles. 

Van Heyden et al. [243] evaluated the performance of AlPO-18 coated on aluminium supports with polyvinyl alcohol (PVOH) as a binder for heat exchanger applications by measuring the kinetics of water adsorption onto AlPO-18. The AlPO-18 layers showed the decreased heat and mass transfer characteristics with increasing the thickness and crystals size (from nano- to micron-sized). However, with diffusion limitation of microporous AlPO taken into consideration, they concluded that a critical layer thickness was about 200 µm. Below this limit value, fast heat exchange cycles were observed thereby allowing for appropriate heat transformation. Furthermore, in view of potential uses as adsorbents in the thermochemical energy storage operating as open and closed storage systems, the effective thermal conductivity of AQSOA FAM-Z02 packed bed adsorbers was modelled as a function of such physical factors as water uptake, number of adsorbent layers, particle size, bed porosity, temperature, contact pressure, and interstitial gas pressure. The model was confronted with the experimental results achieved by means of heat flow meter method by Rouhani et al. [244]. The effective thermal conductivity of the 2 mm-size FAM- Z02 was found to lie between 0.188 (at 283 K) and 0.204 W m^−1^ K^−1^ (at 353 K). With the 0.32 g g^−1^ of water uptake at 303K, the 2 mm FAM-Z02 open system displayed the effective thermal conductivity 2.2 times higher than that measured for the closed system, i.e., 0.1031 against 0.0474 m^−1^ K^−1^. Based on this model, an optimum particle size could be estimated for any given bed thickness in order to achieve the highest thermal conductivity.

### 5.4. Metal-Organic Framework Structures (MOFs)

Owing to their well-characterized crystalline structure and high specific surface areas, metal-organic frameworks (MOFs), also called porous coordination polymers, constitute another class of synthetic porous materials of interest for uses in adsorption, separation, heat storage, and catalysis [245]. 

MOFs are crystalline inorganic-organic hybrid materials, comprising single metal ions or polynuclear metal clusters connected by organic linkers with a one, two or three- dimensional framework through coordination bonds. With different organic linkers and metal centre combinations, there exists practically an endless possibility of various structures and a high chemical versatility. The nanosized cavities and/or open channels formed by the two or three-dimensional framework of MOFs provide enormous potential for adsorption applications.

During the last two decades, tremendous efforts have been made to the design and synthesis of MOF materials. These materials have been developed through four different generations [246,247]. The first three generations were described in the work of Kitagawa and Kondo [248]: the first generation materials unfortunately collapsed after the removal of guest (adsorbates) and they lost the crystallinity and porosity; the stability was reinforced in the MOFs of second generation possessing rigid frameworks even upon guest (adsorbates) removal; the third-generation MOFs were characterized by a great flexibility of their frameworks due to temperature and pressure or upon adsorption of gas molecules (e.g., thermal and guest-induced structural transformations between the Large (LP) and Narrow Pore (NP) forms of the MIL-53 materials referred to as “breathing” phenomenon [249,250]). The fourth-generation MOFs have been achieved by means of the post-synthesis modifications, recently developed to adjust pore size and surface chemistry so as to accommodate different guest species without losing the inherent topology and structural integrity [246,247,251,252]. More recently, MOFs with complex systems containing defects or being non-stoichiometric (Solid Solution) [246], were included into the latest fourth generation, together with other types of MOFs combining a rigid framework with self-switching pores that could adapt themselves to a particular guest through the rearrangement of extra-framework counter-ions or reorientation of flexible linkers.

The conventional synthesis of MOFs is usually carried out by following the “one-pot” procedure involving either slow diffusion or direct mixing of metal ions and organic precursors under solvothermal conditions [253,254]. The main advantage of the synthesis of MOFs in comparison with other template-assisted preparation procedures (e.g., SAPO-34) is strictly related to the use of appropriate solvent which itself acts as the main template and for which high-temperature calcination is not necessary to remove it so as to generate the porosity [88]. While conventional synthesis methods of MOFs have relatively matured, Stock and Biswas reviewed such other approaches as mechano-, sono- and electrochemical, or microwave-assisted procedures, as well as high-throughput synthesis [253]. These methods have shown to be applicable to certain compounds, often under milder reaction conditions, thus yielding materials possessing different particle sizes and other properties. Further tuning of the material’s properties by introducing functional groups into MOFs, covering covalent and dative modifications, as well as post-synthesis deprotection of functions, were reviewed by Cohen et al. to emphasize the progress in the post-synthesis modification of MOFs [251,252,254].

Given the numerous studies previously and actually made on MOFs, the present review is focused on some selected cases related to the sorption of water vapour being of potential interest for thermochemical storage.

#### Surface Reactivity and Hydrothermal Stability of MOFs in View of Their Uses as Water Vapour Adsorbents

A lot of experimental and theoretical studies including adsorption measurements or simulations have been carried out to illustrate the capacity of MOFs to adsorb water vapour [255,256,257,258,259,260,261,262,263]. The subject has been already described in several review articles, where specific criteria are given for the selection of MOFs as adsorbents [70,88,264,265,266].

Henninger et al. [88] considered the use of some MOFs in Adsorption Heat Pumps by determining their water uptake capacity, heat of adsorption and cycle stability. Canivet et al. [263,264] proposed two important indicators to evaluate the adsorptive properties on the basis of adsorption isotherms, namely the capacity of water adsorption (in cm^3^ of water per g of sample) and the relative pressure α = p/p_0_ at which half of the total water adsorption is reached. The α parameter was regarded as a measure of the hydrophobic-hydrophilic surface character, independent of the water adsorption capacity. Indeed it increased upon increasing the hydrophobic character and showed an inflexion point in the case of type V adsorption isotherms. De Lange et al. [70] also used α factor to differentiate among various samples, including the uptake in- and outside of the operating window, in parallel with the enthalpy of adsorption and the pore volume as other comparators. They additionally took into account the hydrothermal and cycling stability in view of uses in adsorption heat pumps. A more systematic study of the water stability in MOFs, including both the thermodynamic and the kinetic stability, was made by Burtch et al. [266]. Like in the work of Furukawa et al. [265], the emphasis was put on the condensation pressure of water vapour within the pores, in addition to water uptake, cycling performance and water stability. Although a steep water uptake at low relative pressures may be of interest in dehumidification or water capture systems, its use for the purpose of thermochemical storage was criticized since the extra energy input for conservation and more rigorous regeneration conditions were required.

Canivet et al. [264] identified the following three main mechanisms of water adsorption onto MOFs: (i) the uptake of water molecules on metallic clusters altering the first coordination sphere of the metal ion (chemisorption), (ii) reversible adsorption in the form of layers or clusters, and (iii) irreversible capillary condensation. De Lange et al. [70] proposed three different types of sites for the formation of water clusters: (i) coordinatively unsaturated sites (or unsaturated metal centres) on the metal ions after solvent removal, (ii) terminal hydroxyl groups on the metal-ions of the cluster, when occurring in the system (iii) additional nucleation sites providing hydrophilic functional groups attached to the organic linkers.

One of the first discovered 3-dimensional microporous MOFs, HKUST-1 or Cu-BTC [267,268] (commercialized as Basolite C300 by BASF) has been tested for water adsorption since last decade [86,229,268,269,270,271,272,273,274,275,276]. In the early studies, the unstable framework in the presence of water over several gas sorption-regeneration cycles made this MOF material unsuitable for the storage purposes. Its building unit contained two central Cu^2+^ ions coordinated by four trimesate molecules through their carboxylate groups to form the paddlewheel-like structure of copper acetate Cu_2_(CH_3_COO)_4_(H_2_O)_2,_ with a Fm3m symmetry, and the main pore diameter of 0.9 nm [86,267,275]. Li and Yang first reported a water vapour uptake of 0.26 g g^−1^ onto HUKST-1 at 298K and a suitable hydrothermal stability until p/p_0_ of 0.7 [270]. The framework integrity was confirmed by XRD studies. Unfortunately, this hydrothermal stability could not be reproduced and the framework degradation upon water adsorption was revealed by other researchers [268,271,272,273]. Al-Janabi et al. studied the mechanism of this degradation and showed that water vapour broke the Cu-BTC bonds (BTC for benzenetricarboxylate), thus leading to the formation of BTC-acid [274]. Strong water-Cu coordination was found to displace the carboxylate bonds of BTC ligands from Cu centres which resulted in irreversible change of the framework. Although the cycle stability was accompanied by a partial degradation, the material showed promising water adsorption capacity for the use in sorption processes. Henninger et al. tested HKUST-1 for heat transformation [86] and estimated its water loading capacity at about 0.324 g g^−1^, which was 2.9 times higher than the adsorption capacity of silica gel [86]. A much higher water uptake of 0.55 g g^−1^ was reported by Küsgens et al. [269]. However, the HKUST-1 sample exhibited a continuous degradation of the framework with increasing the number of cycles [229]. Recently, HKUST-1 was demonstrated to keep its structural integrity under dynamic, non-equilibrium conditions when exposed to low humidity and room temperature (600 Pa and 298 K) for up to 48 h of cyclic operations [270].

Another microporous MOF, namely CPO-27 (also named MOF-74), exhibited interesting adsorptive properties with enhanced hydrothermal stability. CPO-27 may use a variety of metallic centres, such as Ni^2+^, Mg^2+^, Co^2+^, and Zn^2+^. The one-dimensional helical chains of cis-edge-connected metal–oxygen coordination octahedra resulted in honeycomb topology with a channel diameter of 1.1 nm. Water uptake was found to occur primarily at very low p/p_0_ values due to the irreversible adsorption of water molecules on the coordinatively unsaturated sites of the metal incorporated in the structure, the adsorption isotherms being generally of type I [255,268,277,278,279,280,281]. The first water adsorption isotherm for CPO-27(Ni) was measured by Liu et al. [282] and presented a saturation capacity of 32 mmol g^−1^ at 298K. More recently, Elsayed et al. reported a maximum water uptake of 0.47 g g^−1^ by CPO-27(Ni) [277]. Schoenecker et al. obtained an adsorption capacity of 37 mmol g^−1^ onto CPO-27(Mg) based on the measurements of adsorption isotherms [268]. Li et al. made a simulation study on CPO-27(Zn) [255]. They demonstrated that multiple adsorption of H_2_O molecules was possible on Zn sites, thus yielding stable H_2_O clusters at low temperatures. The first adsorbed water molecules could be dissociated only at high temperatures, therefore reducing the sorption cyclability [283,284,285]. Tan et al. [284] related the framework destabilization to the water dissociation within the framework at high temperatures. However, like in the case of zeolites, the strong coordinatively unsaturated sites of CPO-27 required higher temperatures to desorb the bound water molecules. Zr-based UiO-66 was also tested for the adsorption of water vapour. The Zr_6_O_4_(OH)_4_ octahedron was shown to form lattices with 12-fold connection through benzenedicarboxylate(BDC) linker, resulting in a highly packed fcc structure possessing cages with tetrahedral (0.74 nm) and octahedral (0.84 nm) shapes (i.e., tetrahedral and octahedral pores) [286,287]. As in the case of microporous zeolites, the adsorption of water vapour was demonstrated to generally follow a pore-filling mechanism. However, UiO-66 appeared more hydrophobic and produced a two-step adsorption isotherm. The first loading step was shown to lie in a p/p_0_ interval of 0.2–0.4, in line with the filling phenomenon within tetrahedral and/or octahedral pores. The second step at p/p_0_ > 0.8 was due to interparticle condensation [288]. One important advantage of UiO-66 was that its adsorptive property and stability could be tuned by post-synthesis modification via direct ligand substitution [287]. A maximum water loading of 0.4 g g^−1^ was found by Jeremias et al. [288], with the average heat of water adsorption being equal to 41.3 kJ mol^−1^. Amino-functionalized UiO-66(Zr)-NH_2_ yielded a more interesting average heat of water adsorption of 89.5 kJ mol^−1^. Unfortunately, it lost 38% of the water adsorption capacity after 40 successive adsorption-desorption cycles. On the contrary, Decoste et al. [289] observed no degradation of UiO-66(Zr) and UiO-66(Zr)-NH_2_ when exposed to water vapour at room temperature. 

Among the most studied MOFs for the water adsorption applications, mesoporous and hydrothermally stable mesoporous MIL-100(Cr) and MIL-101(Cr) outstand from the others because of their high water sorption capacities and low regeneration temperatures. They were first synthesized, studied, and named by Ferey et al. [290]. Their structure consisted of super-tetrahedra (ST) building units, which were formed by rigid terephthalic or trimesic acid linkers and trimeric chromium (III) oxide octahedral clusters, with cavity diameters of 0.29 nm and 0.34 nm. Isostructural compounds with Fe^3+^ or Al^3+^ instead of Cr^3+^ could also be obtained [291]. A high water uptake of 1.01 g g^−1^ onto MIL-101(Cr) was reported by Ehrenmann et al. who obtained an S-shaped adsorption isotherm [89]. Higher water uptake of 1.2 g g^−1^ and 1.47 g g^−1^ were measured, respectively, by Akiyama et al. and Elsayed et al. [277,292]. MIL-101(Cr) exhibited a steep retention of water vapour in a relative pressure range between 0.30 and 0.6 [89]. This hydrophilic-hydrophobic switching behaviour was considered as making the regeneration easier under 363 K. MIL-100(Cr) possessing smaller pores of 2.5 nm and 2.9 nm, was shown to adsorb less water vapour (ca. 0.5–0.8 g g^−1^) due to smaller pore volumes [26]. Recently Cui et al. constructed a MIL-100(Fe) coated heat exchanger with a silica sol binder and tested it in the high temperature cooling system [293]. The theoretical power density was evaluated at 82 W L^−1^ of air. The mechanism of water adsorption onto MIL-100(Cr) and MIL-101(Cr) was studied by De Lange et al. who applied both the experimental and the simulation approach [261]. Water-MOF and water-water interactions were demonstrated to control the adsorption process: at low water loadings, prior to the saturation of all coordinatively unsaturated sites of Cr, the adsorbent-adsorbate interactions determined the shape of the adsorption isotherm; at higher loadings, adsorbate-adsorbate interactions became dominant. Some loss of water adsorption capacity upon cycling was recorded by Ehrenmann et al. (1.9% over 20 cycles and 3.2% over 40 cycles compared to the initial amount of water vapour retained by the MOF framework) [89]. Because of the presence of mesopores having diameters of 2.9–3.4 nm, capillary condensation occurred at undesirably high relative pressures, thus resulting in an adsorption-desorption hysteresis loops. An effort was made to tune the hysteresis loops by functionalizing the MOF structure to obtain MIL-101(–NO_2_), MIL-101(–NH_2_), or MIL-101(–SO_3_H) analogues [292,294]. By varying the hydrophobic character of the functional groups introduced into the linker moiety, more suitable water sorption behaviour could be obtained in view of heat storage uses. 

MIL-53 was another material of this family tested for water adsorption. Unexpectedly, this microporous MOF was also shown to display a hysteresis loop in the adsorption-desorption isotherm [260,295,296]. This particular sorption behaviour was ascribed to the irreversible change in the framework flexibility induced by the sorption of some guest molecules, coinciding with the hysteresis loop. Salles et al. [260] concluded that the “breathing” effect was paralleled by a modification of the hydrophobic-hydrophilic character of the MIL-53(Cr) surface. Although the water adsorption capacity of MIL-53 was only about 0.2 g g^−1^ in comparison with that of MIL-100 or MIL-101, it offered an alternative to tuning the sorption properties towards water vapour by functionalization of the framework with either hydrophobic or hydrophilic groups.

An interesting property of Prussian Blue Analogues was reported quite recently by Boudjema et al. [297]. While the completely dehydrated material showed a hydrophobic surface behaviour, a switch to a hydrophilic character was induced by overcoming the water relative pressure threshold at p/p_0_ ~ 0.03. It is worth noting that much interest has been devoted lately to new MOF materials, which currently constitute one of the most intensively studied adsorbents in the literature. For example, a newly discovered robust large-pore zirconium carboxylate MOF, called MIP-200, was described by Wang et al. [298]. It was found to produce S-shaped adsorption isotherms with a water uptake of 0.39 g g^−1^ below p/p_0_ = 0.25. It was characterized by easy regeneration and good cycling, as well as a notably high coefficient of performance of 0.78 for refrigeration at a low driving temperature below 343 K. 

### 5.5. Other Adsorbents and Adsorbates

Besides the four classes of adsorbents detailed in the previous sections, other materials have been considered for the purpose of adsorption of water vapour and they are discussed below.

Activated alumina (Al_2_O_3_) has been widely used in the moisture capture and catalysis processes [122,299,300,301,302,303]. Commercial activated alumina is generally synthesized by thermal dehydration or activation of aluminium trihydrate, Al(OH)_3_, and the specific surface area depends on the pre-treatment temperature [157,304]. As early as 1971, Carruthers et al. studied the adsorption of water vapour onto various forms of alumina [305]. They revealed five different mechanism schemes which may be followed: 1) H-bonding between water molecules and surface hydroxyl groups (i.e., aluminols), 2) surface hydration of the exposed surface cations by water molecules, 3) dissociative chemisorption in the case of α-alumina, 4) hydration in depth of poorly ordered Al^3+^, originally solvated and not fully coordinated in the oxide structure, 5) hydroxide or oxide-hydroxide formation in depth, e.g., rehydration of transition alumina. Marcussen investigated the kinetics of water adsorption onto alumina based on his model which included a nonlinear adsorption isotherm and simultaneous resistance to mass transfer in the pore system of the solid and in a film surrounding the solid particles [303]. Given a good agreement between the theoretical and experimental data, an effective diffusion coefficient in the pores of 3.6 × 10^−6^ m^2^ s^−1^ and a gas film resistance of 0.85 Re^−0.5^ were determined. Close and Pryor made theoretical study on packed beds containing activated alumina for uses in energy storage units [299,306]. Although activated alumina showed a significant advantage over gravel beds in terms of thermal losses, it was demonstrated to have poorer performance when compared with silica gel beds. Shi et al. summarized 14 models for isotherms of water adsorption onto activated alumina [301]. Desai et al. pointed out that the choice of the regeneration temperature for the water-loaded alumina depended critically on the desired humidity level [307]. A lower regeneration temperature was sufficient at relatively high humidity, as it was neither necessary nor desirable to remove the chemisorbed water. Serbzov et al. measured isotherms for the adsorption of water vapour onto activated alumina F-200 at 278, 288, 298, and 308 K in the range from 0 to about 95% of relative humidity and obtained a maximum water adsorption of about 25 mmol g^−1^ [122,308]. Higher temperatures under vacuum conditions were needed to completely regenerate the adsorbent. Ferreira et al. compared the adsorption capacity of 13X zeolite, activated alumina and pure silica towards water vapour and carbon dioxide from air on the basis of the equilibrium adsorption isotherms at 308 K [309]. They found that 13X exhibited the highest adsorption capacity towards both adsorbates at low relative pressures. The S-shape of adsorption isotherms in the case of activated alumina (maximum amount adsorbed of 4.55 mol kg^−1^) and silica (2.66 mol kg^−1^), with an inflection point at an intermediate relative pressure, gave these materials an important advantage over zeolite within the interval of higher relative pressures. Furthermore, the cycling stability of activated alumina was worse compared to silica. This instability was confirmed by Knez and Novak [111] since alumina aerogel was shown to lose almost half of its water adsorption capacity (from 1.2 to 0.6 g g^−1^) after 10 adsorption-desorption cycles.

Clay materials have been investigated in heat storage owing to their large specific surface areas, low cost and high availability. Their surface area ranges between 300 and 700 m^2^ g^−1^ and they are known for their ability to adsorb much water in quantity corresponding to about 7–10 times their volume [310], thus resulting in strong expansion (swelling) of the interlayer space. Clays are built of negatively charged aluminosilicate layers kept together by some compensating cations [311,312,313,314,315]. Heat of adsorption can be released upon contact with water vapour through the penetration of water molecules between the layers due to hydrogen bonding with the hydroxyl groups present in the clay structure and also through the hydration of the exchangeable cations [311,314]. Such “interlayer swelling” depends on the nature of clay and that of compensating cations. 

Hydration-dehydration behaviour of bentonite, consisting mostly of montmorillonite, has been widely studied in view of heat storage uses [310,313,316,317,318,319,320]. Konta compared bentonites with different compensating cations, such as Ca^2+^ and Na^+^ [311], and concluded that Na-bentonite tended to be highly dispersed in water, whereas Ca-bentonite showed a tendency to coagulate. Sadek and Mekhemer [310,316] argued the suitability of using Ca- and Na-montmorillonite clays as thermal energy storage materials. With pre-heating at 398 K, the adsorption capacity towards water and energy storage reached the following values: 0.25 g g^−1^ and 1360 J g^−1^ (i.e., 435 cal g^−1^) for Ca-montmorillonite; 0.29 g g^−1^ and 2843 J g^−1^ (i.e., 679 cal g^−1^) for Na-montmorillonite, respectively. They indicated that the compensating cation, its size and its ionic charge played a significant role in determining the capacity of storing thermal energy. Salles et al. [312] confirmed that the driving force for hydration of the montmorillonite type clays was generally a function of the nature of the interlayer cation. Furthermore, the presence of two types of cation (e.g., Na^+^ and Ca^2+^) may produce the heat release stages at various values of relative humidity, which could be of interest for storage uses [321]. Finally, Castrillo et al. [322] demonstrated that the regeneration of bentonite at low temperatures (below 373 K) was not 100% effective, leading to a slow decrease in the adsorption capacity after successive regeneration steps (i.e., loss of 15–17% after 5 cycles). 

Activated carbons have also been widely used as adsorbents owing to their large micropore and mesopore volumes, as well as their high surface areas ranging between 300 and 4000 m^2^ g^−1^. The manufacturing process usually involves: (i) raw material preparation, (ii) low-temperature carbonization, and (iii) activation procedures [157]. The main specificity of activated carbon is related to its weak polar character mainly in relation with the presence of surface oxide groups, various heteroatoms inserted in polyaromatic rings or inorganic impurities. When water molecules are adsorbed on the carbonaceous surface at low vapour pressures, the direct adsorbent-adsorbate interactions are of the van der Waals type. As a consequence, isotherms of water adsorption represent type V curves. At higher vapour pressures, clusters of water molecules are formed and eventually pore filling occurs through hydrogen bonding [323].

Experimental study of water adsorption on activated carbons at low surface coverage ratios led Salame and Bandosz to conclude on the dependence of water sorption on the surface chemistry and porosity of the adsorbent [324]. A significant contribution to the isosteric heats of adsorption due to water-water interactions was recorded even at very low relative pressures. Huber et al. proposed a monolithic nitrogen-doped carbon as a water sorbent for adsorption refrigeration technology [325]. This carbonaceous sample was prepared from a resorcinol-melamine-formaldehyde resin moulded into monolithic shapes before pyrolysis and chemical activation with KOH. At the 2300 Pa pressure of water vapour, the specific cooling power arrived at 192 W kg^−1^ of water vapour for a temperature decrease from 363 K to 323 K and 389 W kg^−1^ when the temperature passed from 333 K to 303 K. This cooling performance was higher than that of commercial silica gel. Given the weak polarity of carbon surface, other adsorbates, less polar than water, were adsorbed onto activated carbon. For example, Critoph demonstrated the potential of methanol and ammonia as adsorbates for small solar-powered refrigerators [326]. Nevertheless, the use of such adsorbates should be limited to closed systems for security and health reasons. 

The adsorption of methanol or ethanol onto MOF materials may be also of interest for the storage applications. De Lange et al. studied the adsorption of methanol or ethanol as the working fluid onto 18 different MOF structures for uses in adsorption-driven heat pumps and chillers [327]. They arrived at the following conclusions, in comparison with water vapour employed as the adsorbate: (i) adsorption occurred at lower relative pressures in the case of methanol and even much lower for ethanol, (ii) larger pores could be utilized more efficiently, since the hysteresis loops were absent until the pore size attained at least 3.4 nm (only 2 nm for water), (iii) larger pore sizes were needed to ensure the desired stepwise adsorption, (iv) the impact of functional groups in the MOF framework was far less pronounced, (v) the energy released or stored up per one cycle was lower, but the heat and mass transfers were enhanced, (vi) the framework stability would be less of an issue, with notable exceptions of UiO-67 and CAU-8.

MIL-100 and MIL-101 were not appropriate for the present uses because of the high temperatures required for desorption in relation with the type I adsorption isotherms. From the thermodynamic viewpoint, UiO-67, CAU-3, and ZIF-8 samples appeared more adequate for adsorption of methanol and ethanol. Because of moderate desorption temperatures, these materials could outperform activated carbons. Even though UiO-67 was not completely stable during adsorption of ethanol and methanol, both CAU-3 and ZIF-8 showed great potential for applications, especially in sub-zero temperature adsorption chillers. The choice of methanol (higher energy capacity) or ethanol (higher temperature lift) was found to depend on the evaporation temperature required for a given application.

## 6. Concluding Remarks

Amongst various concepts considered in the scientific and technical literature to harvest solar energy during summer season and store it until wintertime in view of space heating uses, low-temperature thermochemical storage based on solid-vapour adsorption seems to constitute a promising alternative because the storing principle relies on quite well-controlled changes in the chemical potential of a solid material and is far less sensitive to temperature variations between the charging and discharging steps. Contrary to thermal storage methods using sensible or latent heat, the charging (i.e., endothermic vapour desorption from the adsorbent surface) and discharging (i.e., exothermic vapour adsorption onto adsorbent) temperatures do not need to be the same and the energy storage density depends to a much smaller extent on the discharging (adsorption) temperature. Open sorption systems operating in the *moist-air flow mode* offer the advantage of operating under atmospheric pressure and ambient temperature, thereby avoiding potentially detrimental conditions of high partial pressures of adsorbate in combination with elevated temperatures. Nevertheless, the thermochemical storage technology is still at too low maturity level, the only industrial implementation known nowadays corresponds to a short-term (day-night cycle changeover) heat storage unit using zeolite 13X beds to adsorb water vapour and thus heat a school building. The selection of materials should be carefully adapted to real operating conditions and this is probably the main reason that has continuously motivated the researchers to propose new adsorbents performing under particular conditions of use.

**Figure 5:**
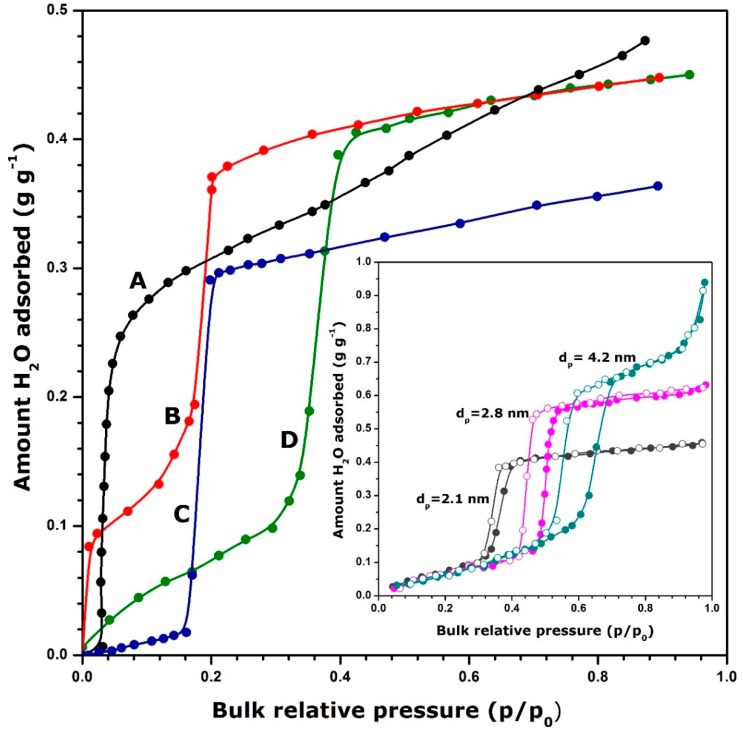
Examples of S-shaped adsorption isotherms for water vapour onto different adsorbents at 298 K (with the exception of Prussian Blue Analogue and MIP-200: data at 303 K): Prussian Blue Analogue (Curve A); zeotype silico-aluminophosphate, MIP-200 (Curve B); Metal Organic Framework, CAU-10 (Curve C); ordered mesoporous silica of the MCM-41 type (Curve D). The amount of H_2_O adsorbed expressed as mass of the adsorbate per unit mass of the adsorbent. The inset shows the effect of the pore size on the location of the adsorption (solid circles) and desorption (open circles) isotherms obtained with ordered mesoporous silicas of the MCM-41 type. Data adapted from [172,297,298,328].

In order to avoid creating or aggravating problems related to the adsorbent regeneration (application of vacuum and high-temperature degassing processes) or long-term storage of the adsorbent between the charging and discharging steps without loss of its activity (use of vacuum-tight tanks), materials exhibiting a very hydrophilic surface character and strongly adsorbing much water vapour already at low vapour pressures are not necessarily the best candidates for low-temperature heat storage by adsorption of water vapour. The adequate adsorption isotherm should rather exhibit a particular, stepwise shape, as those exemplified in Figure 5. In such systems, even if all adsorbed water is not released from the solid surface during the charging step, the increase in the water uptake in the intermediate pressure region may be sufficiently high for the adsorbent to be of interest for use in heat storage. Hence the relative vapour pressure employed during the discharging step (e.g., the humidity level in the carrier air flow) has to be high enough to reach the adsorption plateau value. It is thus clear that the location of the quasi-vertical portions of the adsorption and desorption isotherms should be chosen in line with the operating conditions. The inset graph in Figure 5 additionally illustrates the fact that this location is not constant for a given type of solid material and may, for example, depend on the pore size, which makes the adsorbent selection less evident.

Synthetic materials possessing uniformly structured porosity with high specific surface areas and tuneable or switchable hydrophobic-hydrophilic surface character seem to meet the above requirements and they will certainly constitute the adsorbents of the future. This may be the case of certain ordered mesoporous silicas, microporous MOF materials, or Prussian Blue Analogues, provided that they meet the requirements of hydrothermal stability, shaping and up-scaling. Moreover, much more effort will be necessary to further assess the performance of selected adsorbents on pilot scale with simulated operating conditions before considering their use in full-scale applications in thermochemical storage of energy by sorption.

**Figure 6:**
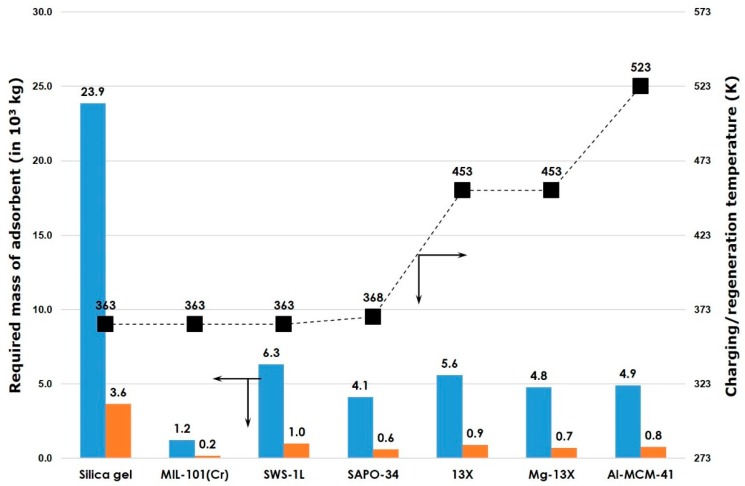
Assessing the efficiency of thermochemical storage by adsorption of water vapour onto selected adsorbents: simulation of the mass of adsorbent required to cover, during 22 winter’s coldest days, the energy needs for space heating in a 100 m^2^ house located in the Parisian (North of France, in blue) and Marseille (South of France, in orange) regions which comply with the new RT2018 standards, namely 3000 MJ (i.e., 836 kWh) and 460 MJ (i.e., 128 kWh), respectively, to maintain the indoor temperature at 292 K [329]. The energy density and charging/regeneration temperature data have been taken from [23,69,87,92,112].

In light of the discussion on the properties of materials considered in the present review, it seems appropriate to reflect on the potential effectiveness of the thermochemical storage by sizing the process for space heating in the building sector. Figure 6 presents the results of simulation of the mass of various adsorbents required to cover, during 22 winter’s coldest days, the energy needs for space heating in 100 m^2^ houses located in the Parisian (North of France) and Marseille (South of France) regions which comply with the current RT2018 standards. It is important to realise that the energy input during this reference period accounts for almost one third of the total annual needs for space heating.

It is clear from the figure that the principle of thermochemical storage by water adsorption onto porous adsorbents is not capable of covering alone the energy needs for space heating in homes, small businesses and public buildings if the quantity of the working materials employed should remain within reasonable limits. The phenomenon may be rather exploited in the auxiliary heating systems, which provide additional heat on just the coldest days, for example during 22 “EJP” peak tariff days set by the French electricity provider EDF [329,330].

## Figures and Tables

**Table 1:** Physical properties of representative materials for thermal and thermochemical energy storage illustrating their storage performances [22,23,24].

Physical Property or Characteristics	Heat Storage Principle and Working Materials					
	Sensible Heat Storage		Latent Heat Storage		Adsorption Heat Storage	
	Water	Rock	Paraffin Wax	CaCl_2·_6H_2_O	Silica Gel/Water	Zeolite/Water
Latent heat of fusion (kJ kg^−1^)	-	-	174.4	160	-	-
Specific heat capacity (kJ kg^−1^ K^−1^)	4.18	0.9	-	-	1.13	1.07
Heat of adsorption (kJ kg^−1^ solid)	-	-	-	-	1380	1107
Density (kg m^−3^)	1000	2240	1802	1830	600	650
Volume of material for storing 1GJ (m^3^)	4.8	9.9	3.2	3.4	1.2	1.4
Heat storage density (MJ m^−3^ solid)	209	100	310	292	767	713
Advantages	(1) simplicity of design and use (2) low implementation costs		(1) lower heat loss		(1) higher storage density (2) smaller volume of working materials (3) very low heat loss (4) lower charging and discharging temperatures	
Weaknesses	(1) low energy density (2) high heat loss (self-discharging effects possible) (3) space limitations (large volume of working materials)		(1) low thermal stability (risk of chemical decomposition at high temperatures) (2) costly investment in thermal insulation		(1) complexity of design and use (2) higher investment costs (3) heat and mass transfer limitations	

**Table 2:** Physical and chemical properties of some selected substances encountered in thermochemical heat storage by adsorption at the Solid-Gas interface [66,78,79,80,81].

Substance	H_2_O	NH_3_	CO_2_	C_2_H_5_OH	CH_3_OH	N_2_	O_2_	He	Air (Dry)
Critical temperature (K)	647	405.3	304	513.9	512.4	126	154.4	2	132.5
Boiling point at 10^5^ Pa (K)	373	239.7	194.5	351.3	337.6	77	90	5	-
Enthalpy of vaporization (kJ mol^−1^)	40.65	23.4	15.3	38.56	35.21	5.57	6.82	0.08	-
Saturated vapour pressure at 293 K (kPa)	2.34	857.1	5.7∙10^3^	5.8	12.8	-	-	-	-
Isobaric heat capacity in the gas phase at 300 K and 10^5^ Pa (J mol^−1^ K^−1^)	33.6	37.0	36.94	74	44.1	29.2	29.4	20.8	9.15
Thermal conductivity in the gas phase at 300 K and 10^5^ Pa (mW m^−1^ K^−1^)	18.7	24.4	16.8	14.4	26.2^400 K^	25.8	26.3	156.7	26.2
Kinetic diameter of molecules (nm)	0.265	0.260	0.330	0.450	0.360	0.364	0.346	0.260	-
Dipole moment for molecules in the gas phase (in debyes)	1.85	1.47	0	1.69	1.7	0	0	-	-
Proton affinity (kJ mol^−1^)	697	853.5	548	788	761	495	422	178	-
Absolute hardness parameter (eV)	9.5	8.2	8.8	8.0	5.8	8.9	5.9	-	-

Flammability: Water and carbon dioxide are not flammable and cannot be ignited; the flammable range of NH_3_, C_2_H_5_OH, and CH_3_OH in air is (in percent by volume): 15–28, 3–19, and 6–37, respectively [82]. Toxicity: Ammonia vapours are highly toxic through inhalation and lead to irritation of the skin, eyes and respiratory tract [83]; they are corrosive upon contact and their use with copper or its alloys is to be avoided. Carbon dioxide (at concentrations in air greater than 10%) presents both acute and long-term toxicity with respect to the lungs, the cardiovascular system, and the bladder, showing inflammatory and possible carcinogenic effects, as well as it may induce multiple foetal malformations [84].

**Table 3:** Selected groups of materials considered for adsorption-based low-temperature thermochemical storage of energy.

Adsorbent Group	Working Couple	Textural Properties of Adsorbent		Adsorption Performance		Storage Performance Tests			Ref.
		Porosity Type	Specific Surface Area (m^2^ g^−1^)	Capacity (g g^−1^)	Heat (kJ mol^−1^)	Target Storage Density (kJ kg^−1^)	Operating Conditions	Information on Stability	
Amorphous SiO_2_	silica aerogel & H_2_O	micro-/meso-	783	1.35	-	-	*charging:* vacuum, 343 K, 24 h *discharging:* 293 K	Adsorption greatly decreasing after the first cycle (raw sample: 42%; calcined one: 26%), then remained stable over next 25 cycles	[111]
	silica gel LE32 & H_2_O	macro-	-	0.6	25	86	*charging:* 363 K *discharging:* 313 K	-	[23]
Amorphous Al_2_O_3_	alumina aerogel & H_2_O	micro-/meso-	453	1.25	-	-	*charging:* 343 K *discharging:* 293 K	adsorption slowly decreasing within 10 cycles, then remained stable	[111]
	30% alumina + 70% silica aerogel & H_2_O	micro-/meso-	577	1.15	-	-		stable over 25 cycles	
Ordered SiO_2_	MCM41 & H_2_O	meso-	1137	0.04 at p/p_0_ = 0.3	47–54	-	*charging:* 523 K, 3 h *discharging:* 293 K	-	[92]
	SBA15 & H_2_O	meso-	554	0.02 at p/p_0_ = 0.3	-	-		-	
Salt-oxide hybrids	CaCl_2_-slica gel KSK (SWS-1L) & H_2_O	meso-	230	0.7	43.9–63.1	475	*charging:* 353–423 K	-	[112,113,114]
	Ca(NO_3_)_2_-silica gel KSK (SWS-8L) & H_2_O	meso-	60	0.23	47–52	-	*charging:* 348–353 K	-	[115]
	Al-MCM41 & H_2_O	meso-	941–1021	0.17 at p/p_0_ = 0.3	65	450–612	*charging:* 253 K *discharging:* 293 K	-	[92]
	Al-SBA15 & H_2_O	meso-	541–550	0.09 at p/p_0_ = 0.3	67	227–335		-	
Zeolites	4A & H_2_O	micro-	-	0.22	72	670	*charging:* 463 K *discharging:* 298 K	-	[116]
	13X & H_2_O	micro-	-	0.34	51.3	536	*charging:* 723 K *discharging:* 300 K	-	[69]
	MgNa-X & H_2_O	micro-	-	0.212	53	630		-	
	MgSO_4_-13X & H_2_O	micro-	-	0.15	81.6	648	*charging:* 423 K	adsorption unchanged over 3 cycles	[117]
Zeotype materials	AlPO-18 & H_2_O	meso-	-	0.34	55	875	*charging:* 268 K *discharging:* 313 K	-	[87]
	SAPO-34 & H_2_O	meso-	-	0.3	56	730		-	
MOF materials	MIL-101 (Cr) & H_2_O	meso-	4150	1.6	44.5	-	*charging:* 423 K *discharging:* 303 K	stable over 10 cycles	[118]
	MIL-100 (Fe) & H_2_O	meso-	2300	0.87	49.5	-		stable over 10 cycles	
